# Oxidation of the alarmin IL-33 regulates ST2-dependent inflammation

**DOI:** 10.1038/ncomms9327

**Published:** 2015-09-14

**Authors:** E. Suzanne Cohen, Ian C. Scott, Jayesh B. Majithiya, Laura Rapley, Benjamin P. Kemp, Elizabeth England, D. Gareth Rees, Catherine L. Overed-Sayer, Joanne Woods, Nicholas J. Bond, Christel Séguy Veyssier, Kevin J. Embrey, Dorothy A. Sims, Michael R. Snaith, Katherine A. Vousden, Martin D. Strain, Denice T. Y. Chan, Sara Carmen, Catherine E. Huntington, Liz Flavell, Jianqing Xu, Bojana Popovic, Christopher E. Brightling, Tristan J. Vaughan, Robin Butler, David C. Lowe, Daniel R. Higazi, Dominic J. Corkill, Richard D. May, Matthew A. Sleeman, Tomas Mustelin

**Affiliations:** 1 Department of Respiratory, Inflammation and Autoimmunity, MedImmune Ltd, Granta Park, Great Abington, CB21 6GH, UK; 2 Department of Antibody Discovery and Protein Engineering, MedImmune Ltd, Granta Park, Great Abington, CB21 6GH, UK; 3 Department of Biopharmaceutical Development, Analytical Biotechnology, MedImmune Ltd, Granta Park, Great Abington, CB21 6GH, UK; 4 Department of Discovery Sciences, Innovative Medicines, AstraZeneca, Mereside, Alderley Park, SK10 4TG, UK; 5 Department of Respiratory, Inflammation and Autoimmunity, MedImmune LLC, Gaithersburg, Maryland 20878, USA; 6 Department of Infection, Immunity and Inflammation, Institute for Lung Health, Leicester LE3 9QP, UK; NIHR Respiratory Biomedical Research Unit, University Hospitals of Leicester, Leicester LE3 9QP, UK

## Abstract

In response to infections and irritants, the respiratory epithelium releases the alarmin interleukin (IL)-33 to elicit a rapid immune response. However, little is known about the regulation of IL-33 following its release. Here we report that the biological activity of IL-33 at its receptor ST2 is rapidly terminated in the extracellular environment by the formation of two disulphide bridges, resulting in an extensive conformational change that disrupts the ST2 binding site. Both reduced (active) and disulphide bonded (inactive) forms of IL-33 can be detected in lung lavage samples from mice challenged with *Alternaria* extract and in sputum from patients with moderate–severe asthma. We propose that this mechanism for the rapid inactivation of secreted IL-33 constitutes a ‘molecular clock’ that limits the range and duration of ST2-dependent immunological responses to airway stimuli. Other IL-1 family members are also susceptible to cysteine oxidation changes that could regulate their activity and systemic exposure through a similar mechanism.

## Abstract (web summary)

IL-33, released by epithelial cells in response to stress, is a potent activator of inflammation. Here Cohen *et al*. show that secreted IL-33 is rapidly inactivated by disulfide bond formation that prevents binding to its receptor, and that IL-33-related cytokines are susceptible to similar oxidation.

## Graphical abstract

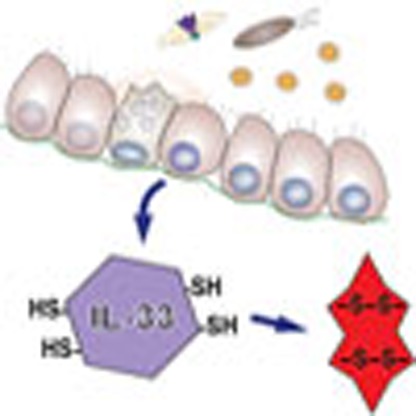

Interleukin (IL)-33 is an IL-1 family alarmin cytokine constitutively expressed at epithelial barrier surfaces where it is rapidly released from cells during tissue injury^1^,^2^,^3^,^4^,^5^,^6^. IL-33 signals through a receptor complex of IL-1 receptor-like 1 (IL1RL1) (known as ST2) and IL-1 receptor accessory protein (IL1RAcP)^7^,^8^ to initiate MyD88-dependent inflammatory pathways. Identification of *IL33* and *IL1RL1* as major susceptibility loci in several genome-wide association studies of human asthma suggests that this axis is likely to play an important role in this inflammatory disease^9^. In support of this, IL-33 has been shown to be upregulated in asthma^10^,^11^,^12^,^13^ and release of IL-33 is increased during disease exacerbation^14^.

Multiple mechanisms have been described to regulate IL-33 activity. Akin to other IL-1 family members, N-terminal processing of full length IL-33 enhances its activity^15^. Conversely, activity at the ST2 receptor can be terminated by caspase cleavage at residue Asp178 within the IL-1-like domain^16^,^17^ or limited via neutralisation by soluble forms of ST2 and IL1RAcP^18^. In addition, IL-33 that binds membrane associated ST2 can be internalized with the receptor^19^,^20^. However little is known about the fate of IL-33 *in vivo* following release from the cell. Here we report a novel mechanism for control of IL-33, namely an oxidation-driven conformational change involving formation of two disulphide bonds, which eliminates ST2-dependent activity. This rapid inactivation of the released IL-33 protein is consistent with its behaviour as an alarmin and serves to limit its range and duration of action. Failure of this mechanism to operate *in vivo* leads to a profound enhancement of inflammation. In addition, the observation that not just IL-33 but many IL-1 family members are susceptible to oxidative changes suggests that this regulatory mechanism may be a common feature of this family of proteins.

## Results

### Oxidation of IL-33 terminates ST2-dependent activity

**Figure 1:**
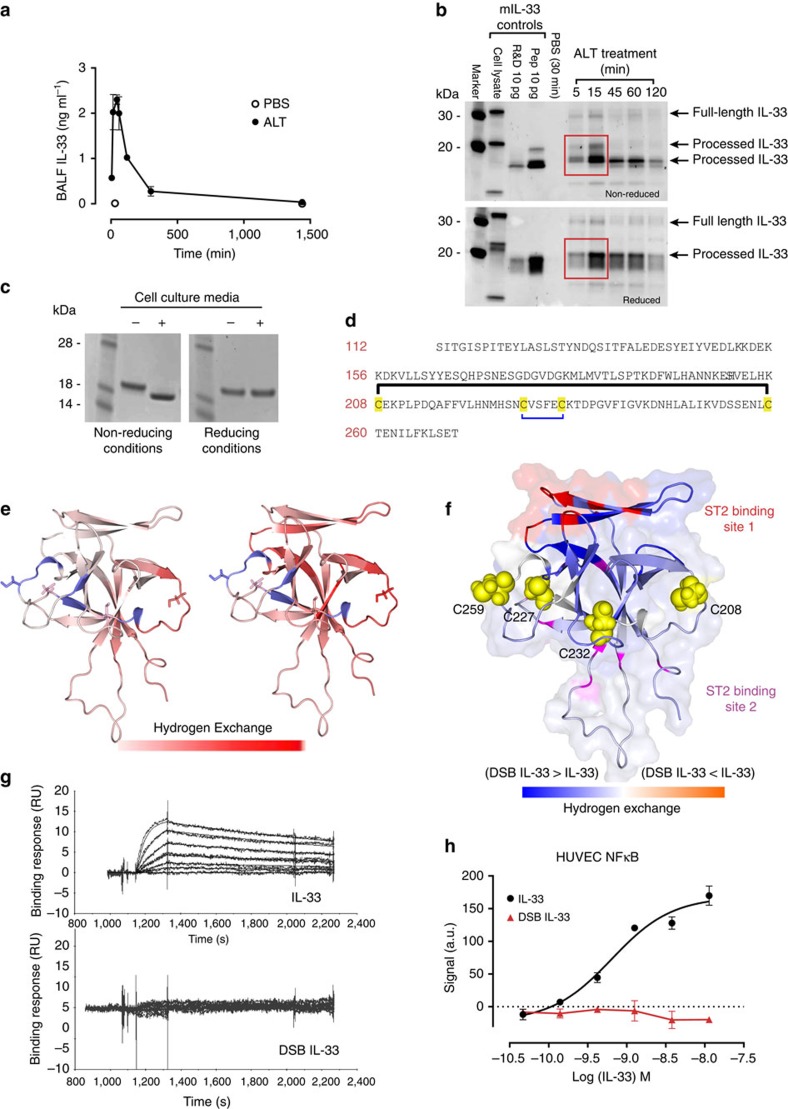
IL-33 is inactivated by disulphide bonding. (**a**) Concentration of IL-33 (mean±s.e.m.) in bronchoalveolar lavage fluid (BALF) following intranasal *Alternaria* (ALT) challenge of BALB/c mice (*n*=3 per group), representative of 2 independent studies. (**b**) Western blot analysis of the same samples (pooled per group) under reducing and non-reducing conditions. Controls are as follows: cell lysate, lysate of HEK cells transfected with full length mouse IL-33; R&D, truncated mouse IL-33 (R&D systems); pep, truncated mouse IL-33 (Peprotech); PBS (30 min), BALF from vehicle (PBS) challenged mice at 30 min timepoint. (**c**) Non-reduced SDS-PAGE of IL-33^112–270^ either untreated (−) or post treatment with cell culture media (Iscoves Modified Dulbeccos Media) (+). Monomeric IL-33 was purified prior to analysis. (**d**) The human IL-33 linear sequence illustrated with the position of the cysteines and the proposed disulphide bridges formed after media treatment. Disulphide mapping was performed on purified monomeric protein. Numbering is based on the full length IL-33 sequence. (**e**) Hydrogen-exchange mass spectrometry (HX-MS) analysis of monomeric IL-33 and DSB IL-33. Comparison of fractional hydrogen exchange (for deuterium) in IL-33 (left panel) and DSB IL-33 (right panel). Data are mapped onto the published IL-33 structure^22^ in both cases for comparison purposes. Gaps in sequence coverage where no HX-MS data could be obtained are highlighted in slate blue. Side chains of cysteine residues are displayed as sticks. (**f**) Structural model displaying the difference in fractional hydrogen exchange between IL-33 and DSB IL-33 overlaid with the ST2 binding sites (red and magenta)^23^. Only increased hydrogen exchange was observed for DSB IL-33 versus IL-33, with regions exhibiting the greatest differences of hydrogen exchange depicted in dark blue. (**g**) ST2 binding of human IL-33 (upper panel) and purified DSB IL-33 (lower panel) measured by surface plasmon resonance. (**h**) Signalling in human umbilical vein endothelial cells (HUVEC) stimulated by human IL-33 and purified DSB IL-33. NFκB p65/RelA translocation was measured at 30 min post stimulation. Data points are mean±s.e.m. of duplicate determinations, representative of 3 independent experiments.

To study the release of IL-33 in the lung, mice were challenged intranasally with the clinically relevant fungal allergen *Alternaria alternata* (ALT)^4^,^21^. Immediately following ALT challenge 1–2 ng ml^−1^ of IL-33 were detected in bronchoalveolar lavage fluid (BALF) samples (Fig. 1a), peaking between 15 and 60 min. The released IL-33 protein in BALF consisted mainly of a ∼19 kDa mature form (Fig. 1b, Supplementary Fig. 1). Only minor amounts of full-length IL-33 (∼30 kDa) were detectable. SDS–PAGE under reducing or non-reducing conditions revealed differences in apparent molecular mass of the processed IL-33, implying the presence of redox-related modifications. Recombinant, N terminally truncated mouse IL-33 proteins used as controls also showed similar changes in migration between reducing and non-reducing gels (Fig. 1b).

We sought to investigate if the ability to undergo redox modifications was also a physicochemical property of human IL-33. Recombinant human IL-33 incubated overnight in cell culture media eluted earlier from a size exclusion chromatography column than untreated IL-33 (Supplementary Fig. 2) and the purified monomer showed a change in electrophoretic mobility detectable by SDS–PAGE run under non-reducing, but not reducing, conditions (Fig. 1c, Supplementary Fig. 3). Analysis of this material by mass spectrometry revealed a 4-Da reduction in mass compared with untreated IL-33 (Supplementary Fig. 4), consistent with the formation of two disulphide bridges. Disulphide mapping confirmed the formation of two intrachain disulphide bonds (Fig. 1d, Supplementary Fig. 5, Supplementary Table 1). Mass spectra are consistent with bonds between C208-C259 and C227-C232, although other species could not be ruled out. On the basis of the solved structure of IL-33 (refs 22, 23), none of the cysteine side chains are in sufficiently close proximity to one another for disulphide bonds to form without substantial conformational change. Indeed, results from circular dichroism (CD) spectroscopy (Supplementary Fig. 6) indicated a different structure for the disulphide bonded form of IL-33 (from here on referred to as DSB IL-33) compared with the reduced form. Far-ultraviolet spectra were consistent with predominantly β-sheet secondary structures as seen previously with this family of proteins^24^,^25^,^26^,^27^,^28^. Spectra were significantly different demonstrating a change in secondary structure in DSB IL-33, relative to reduced IL-33. In addition, near-ultraviolet spectra observed a difference in ellipticity around Trp absorption, consistent with change in environment of the sole Tryptophan (W193), demonstrating changes to tertiary structure in this region between reduced and DSB IL-33. The difference in intensity around 260 nm is consistent with the introduction of additional chromophores from disulphide bond formation. Nuclear magnetic resonance (NMR) analysis also indicated large changes to the IL-33 protein although detailed structural information could not be obtained (Supplementary Fig. 7). Therefore, to elucidate the structural changes further, hydrogen-exchange mass spectrometry experiments were performed. Major differences between reduced and DSB IL-33 forms were localised to the beta barrel ‘core’ of the protein and top beta hairpin loop (Fig. 1e, Supplementary Fig. 8), that include the high affinity ST2 binding site^23^ (site 1, Fig. 1f). Increased hydrogen exchange is indicative of loss of beta sheet structure in these regions on conversion from IL-33 to DSB IL-33. Consistent with disruption of the receptor binding interface, DSB IL-33 did not bind ST2 (Fig. 1g) and failed to induce any detectable ST2-dependent signalling (Fig. 1h). In summary these data suggest that a conformational switch from IL-33 to DSB IL-33 is a mechanism to terminate IL-33 activity and limit the duration of ST2-dependent immunological responses.

### Disulphide bonding of IL-33 occurs in vivo

**Figure 2:**
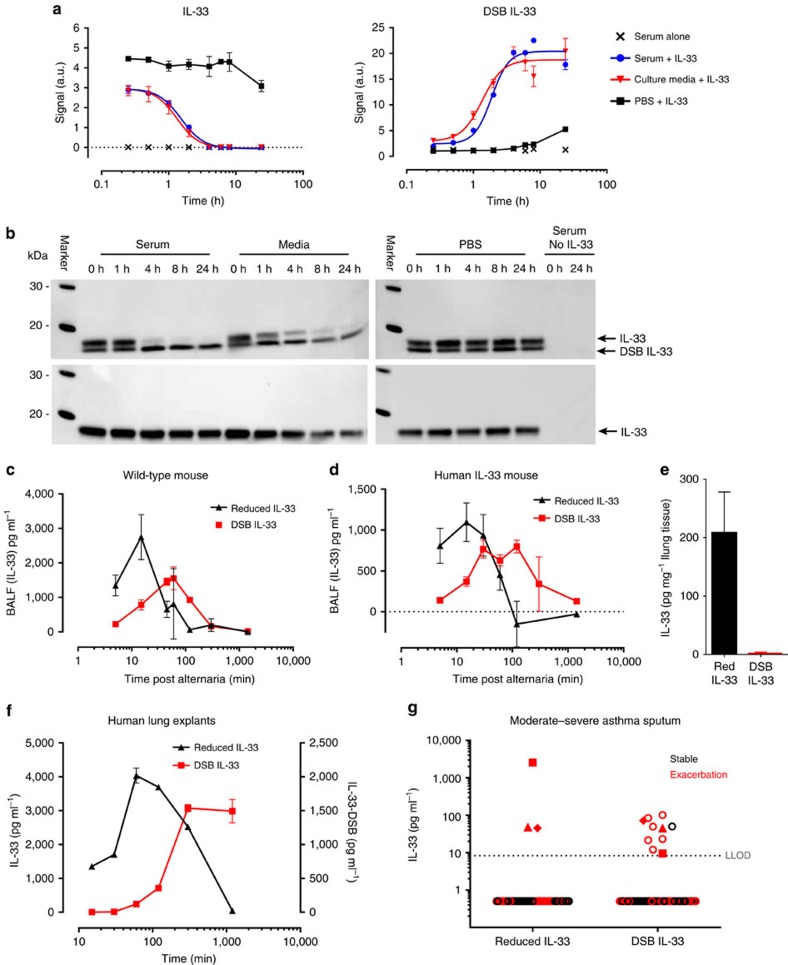
IL-33 converts rapidly to a disulphide bonded form *in vivo*. Change in human IL-33 after exposure to serum or cell culture media measured using (**a**) ELISAs selective for reduced and disulphide bonded (DSB) IL-33. Data points are mean±s.e.m. of duplicate determinations, representative of two independent experiments. (**b**) Western blot of the same samples (pooled per group). (**c**,**d**) Profile of IL-33 forms (mean±s.e.m.) in bronchoalveolar lavage fluid (BALF) following *Alternaria* challenge of (**c**) Wild-type BALB/c (*n*=3 per group) or (**d**) Humanised IL-33 mice (*n*=3 per group). (**e**) Quantitation of IL-33 isoforms in human lung tissue, Mean±s.e.m. for *n*=6 donors. (**f**) Profile of IL-33 forms spontaneously released from human lung explants. Supernatants were pooled from 8 replicates per time point for a single donor and IL-33 levels measured (mean of duplicate ELISA replicates±s.e.m.). Data are representative of 2 individual donors. (**g**) Quantitation of human IL-33 forms in human sputum samples from severe asthmatics. Spontaneous or induced sputum was collected during stable asthma (*n*=37; black) or exacerbation (*n*=35; red) and IL-33 levels measured (mean of duplicate assay replicates). Solid symbols denote samples where both reduced and DSB IL-33 were detectable. Solid symbol shape corresponds to individual subjects. LLOD, lower limit of detection.

To ascertain that DSB IL-33 exists *in vivo*, we used commercially available IL-33 detection assays (Supplementary Fig. 9). We demonstrated that two different ELISAs specific for human IL-33 predominantly detect DSB IL-33. To measure the reduced, biologically active form of IL-33 selectively, we developed ELISAs using capture antibodies that uniquely recognise reduced IL-33 (Supplementary Fig. 10). Collectively, these different assays allowed us to monitor the conversion of IL-33 from reduced to DSB IL-33 *in vitro* and *in vivo*. At 37 °C in culture media or human serum, the reaction occurred rapidly with a 50% conversion in 1–2 h and >90% conversion by 4 h (Fig. 2a). The disappearance of reduced IL-33 correlated well with the appearance of DSB IL-33 and was consistent with changes in gel migration under non-reducing conditions (Fig. 2b, Supplementary Fig. 11).

To study the behaviour and lifecycle of mouse and human endogenous IL-33, we used either wild type (WT) mice or a gene-targeted mouse in which the gene for mouse IL-33 was replaced with the human IL-33 gene (Supplementary Fig. 12). Following ALT challenge, the released IL-33 was predominantly in its reduced form in both WT (Fig. 2c) and humanized (Fig. 2d) mice with a maximum concentration in BALF detected between 5 and 15 min, followed by a rapid decline becoming undetectable by 120 min. Conversely, DSB IL-33 levels gradually increased, peaking at 30–120 min and becoming undetectable by 5 h. Human lung tissue contained high levels of IL-33 (Fig. 2e) that was spontaneously released in explant cultures. Using this endogenous human system we also observed conversion from reduced to DSB IL-33 in human lung explants from COPD patients (Fig. 2f). These data are consistent with a model where IL-33 is released into the extracellular environment in its reduced form, and then rapidly undergoes a conformational switch to a biologically inactive DSB form.

To investigate which forms of IL-33 are present in human disease, we obtained sputum samples from moderate to severe asthmatics during stable disease or during disease exacerbation (Fig. 2g). A positive IL-33 signal was detected in 14% (10/72) of samples and more frequently during acute disease compared with stable disease (9/35 and 1/37, respectively). Non-ST2-binding (presumed DSB) IL-33 was always present (10/10) but reduced IL-33 was detectable only in a subset (3/10). The pattern of data and ratios of reduced to DSB IL-33 in sputum samples are consistent with the changes in IL-33 forms seen in mice and in human lung explants.

### Free cysteines control IL-33 oxidation

**Figure 3:**
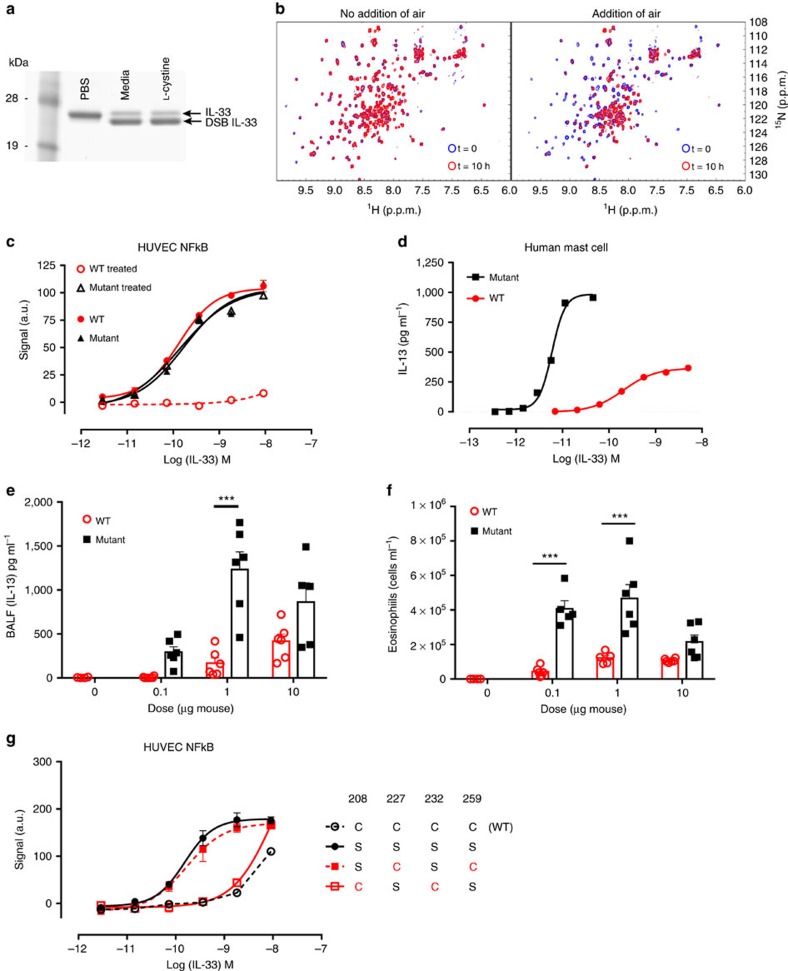
Free cysteines control the conformational switch in IL-33. (**a**) SDS–PAGE of human N-terminal His-Avi tagged IL-33^112–270^ following overnight treatment as indicated. (**b**) NMR analysis with overlay of the ^1^H–^15^N HMQC spectra for ^15^N-labelled human IL-33 following 0 and 10 h incubation in cell culture media without air (left panel) or with air (right panel). Media was degassed before addition of IL-33. No air was added (left panel) or air was pumped through the sample for 5 min (right panel), representative of two separate experiments. (**c**) Signalling in human umbilical vein endothelial cells (HUVEC) stimulated by untreated or media treated human IL-33^112–270^ WT or complete Cys→Ser mutant. NFκB p65/RelA translocation was measured at 30 min post stimulation. Data points are mean±s.e.m. of duplicate determinations, representative of three independent experiments. (**d**) Stimulation of IL-6 production from human cord blood derived mast cells with human IL-33^112–270^ WT or Cys→Ser mutant. Data points are mean±s.e.m. of duplicate determinations, representative of two independent experiments. (**e**,**f**) Concentration of BALF IL-13 (**e**) or eosinophil counts (**f**) following 3 consecutive daily intranasal challenges of BALB/c mice with PBS (*n*=4 per group), human IL-33 WT or Cys→Ser mutant (*n*=6 per group). Endpoint was 24 h after final challenge. Statistical comparisons were made using one way ANOVA with Bonferroni multiple comparisons test (*P<0.05, **P<0.01, ***P<0.001). (**g**) NFκB p65/RelA translocation in human umbilical vein endothelial cells (HUVEC) stimulated by L-cystine treated human IL-33^112–270^ WT or Cys→Ser mutants. Data points are mean±s.e.m. of duplicate determinations, representative of two independent experiments.

*In vitro*, rapid IL-33 inactivation occurred at physiological pH using a disulphide donor L-cystine, a component of cell culture media and serum^29^, (Fig. 3a, Supplementary Fig. 13) and oxygen (Fig. 3b), pointing to an active role for cysteine oxidation in the process. To confirm the importance of the cysteines in the termination of IL-33 activity, we mutated all four cysteines to serine (C208S, C227S, C232S and C259S). This mutant had activity comparable to WT IL-33 (EC_50_ 0.15±0.04 nM versus 0.15±0.02 nM, respectively) in a short duration signalling assay. However, in contrast to the wild type IL-33, the mutant was completely protected from loss of ST2-dependent activity on media or L-cystine treatment (Fig. 3c). The increased stability of this mutant was also apparent in cytokine release assays. Mutant IL-33 was 30-fold more potent than wild-type IL-33 in stimulating IL-13 production from human cord-blood derived mast cells (Fig. 3d) and intranasal administration in mice significantly enhanced IL-13 (Fig. 3e) or eosinophil (Fig. 3f) responses compared with WT IL-33. This is consistent with prolonged activity of the mutant in contrast to the rapid inactivation of the WT IL-33 in the mouse lung environment.

To explore the role of the four individual cysteine residues we made a comprehensive panel of cysteine to serine mutants. Mutation of C208 and/or C232, but not C227 and C259, protected against L-cystine mediated inactivation (Fig. 3g, Supplementary Fig. 14). We then used thiol biotinylation as a means to investigate the relative reactivity of the four free cysteines. We observed that human IL-33 had a tendency to biotinylate on a single cysteine thiol only and mapped this to C208 (Supplementary Fig. 15). C208 is located on the surface of the IL-33 protein, whereas C232 is largely buried^22^. In addition, C208 is within bonding distance of the basic side chain of His195, which could explain the apparent enhanced reactivity of the C208 thiol. We therefore propose that C208 has an initiating role in IL-33 oxidation. Molecular dynamic simulations (Supplementary Fig. 16a) and hydrogen exchange data (Fig. 1e, Supplementary Fig. 8) suggest that the region surrounding C208 is highly dynamic. Furthermore, a monoclonal antibody that binds a linear epitope adjacent to C208 accelerates conversion to the DSB form (Supplementary Fig. 16b,c). Thus, due to its dynamic nature, this region of the molecule may be particularly amenable to local conformational changes that precede disulphide bond formation.

### Other IL-1 family members are susceptible to oxidation

**Figure 4:**
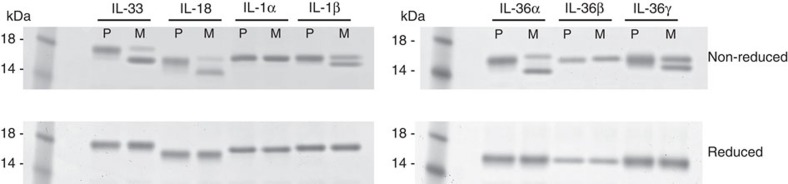
Other IL-1 family members are susceptible to oxidation. SDS–PAGE of mature IL-1 family members following overnight treatment (P, PBS+0.1% BSA; M, cell culture media+0.1% BSA). Non-reducing conditions (top panel); reducing conditions (lower panel).

As other IL-1 family cytokines also contain free cysteine residues we investigated whether there was potential for oxidation to play a role in regulating the mature, secreted forms of IL-1, IL-18 and IL-36. SDS–PAGE analysis indicated that all but IL-1α and IL-36β showed evidence of oxidative changes when treated with cell culture media under the same conditions as IL-33 (Fig. 4, Supplementary Fig. 17).

## Discussion

IL-33 is an alarmin cytokine suggested by genetic association and functional studies to play an important role in inflammatory diseases such as asthma. The solved structure of mature human IL-33 shows that it belongs to the β-trefoil family of proteins and has four free cysteine residues^22^,^23^. In the present study we sought to characterise endogenous IL-33 protein and, in doing so, identified a previously undescribed form of IL-33 containing two disulphide bridges. Here we establish cysteine oxidation as a critical regulatory mechanism *in vivo* for terminating IL-33 cytokine activity at its receptor ST2. We propose that this novel mechanism for the rapid inactivation of secreted IL-33 constitutes a ‘molecular clock’ that limits the range and duration of ST2-dependent immunological responses.

To characterise the endogenous IL-33 protein released in lung, we used ALT challenge to provide detectable quantities of protein. We found IL-33 to be released even more rapidly than described^4^, with maximal levels in BALF at 15 min after challenge (Fig. 1a, Supplementary Fig. 1). In fact, we were only able to visualize distinct redox isoforms by western blot at very early time points not previously studied by other investigators. In our experiments the IL-33 protein released into BALF of WT mice was mainly a ∼19 kDa mature form consistent with reported N terminally proteolytically processed forms^15^. Our data are in contrast with other studies that suggested release of only full length IL-33 following ALT challenge^21^. The reason for this discrepancy is unclear.

We did not see any IL-33 specific bands corresponding to dimer or higher molecular weight species in our *ex vivo* samples, thus the predominant oxidized IL-33 isoform in the lung appears to be monomeric. In contrast, we did see formation of some multimeric IL-33 when oxidation of recombinant IL-33 was performed at high concentrations (Supplementary Fig. 2). To be physiologically relevant and consistent with our *in vivo* observations, we purified and analysed only monomer species and therefore define DSB IL-33 with 2 intramolecular disulphide bonds. However, due to the distance between the cysteines and the rigidity of the β-barrel structures of IL-33, it is clear that significant protein unfolding and conformational change must occur to form the proposed disulphide bridges (Fig. 1d). Consistent with this, data from structural investigations indicate that much of the DSB IL-33 may be relatively unstructured compared with reduced IL-33. Nevertheless, far-ultraviolet CD spectra and hydrogen exchange data, generated using much lower protein concentrations than NMR, both suggest that some β-sheet secondary structures may be preserved in DSB IL-33. Importantly, the regions disrupted in DSB IL-33 contain the high-affinity ST2 binding site, providing a structural explanation for the loss of ST2 binding and signalling for the DSB IL-33 isoform.

Before release, IL-33 stored in human and mouse lung tissues was a reduced, ST2-binding isoform (Fig. 2e). This is consistent with our finding that the form of IL-33 detected immediately after release is also reduced (Fig. 2c,d,f). However, in sputum from exacerbating asthmatics, DSB IL-33 appeared to be the predominant form detected (Fig. 2g). As one acute sputum sample contained almost entirely reduced IL-33, we believe our collection methods were suitable to minimise oxidation during sample handling. In addition, this same sample contained very high IL-33 levels, consistent with being near to the time of IL-33 release in this patient. During an asthma exacerbation, the exact kinetics of IL-33 release are unknown and timing of patient sampling is variable. Therefore, in the sputum data presented here, we feel that the timing of sample collection is the most likely explanation for frequent detection of DSB IL-33.

We did not characterize serum IL-33 in this study. However, our data suggest that any free IL-33 reaching the plasma is likely to be rapidly oxidised (Fig. 2a,b). We demonstrated that commercially available ELISA assays for human IL-33 predominantly detected DSB IL-33. As these assays have been widely used to quantitate IL-33 in human *ex vivo* samples^14^,^30^,^31^,^32^, it appears possible that DSB IL-33 may have been the main circulating species reported to date. However, based on our own observations, studies of tissue (intracellular) IL-33 will likely have measured reduced IL-33 (Fig. 2e). This raises important points for interpretation of existing data. First, depending on the form of IL-33 measured versus that used as a reference standard, IL-33 quantitation in samples may be inaccurate. Second, our data demonstrate the difficulty in validating ELISA systems. Without knowledge of DSB-IL-33 it would be concluded that the assay sensitively measured ST2-bound IL-33. However, it is now clear that the assay can detect two forms of ‘free’ (non-ST2 bound and non-ST2 binding) IL-33. Finally, the challenges of ELISA interpretation identified here are of broader significance if one considers that numerous proteins have various *in vivo* isoforms, variants or binding partners. Therefore, for many proteins there may be multiple species detected to a different extent. Without complete knowledge of these, analyte quantitation and biological interpretation may be compromised.

IL-33 is a member of the IL-1 family of cytokines^33^, which have important functions in host defence and immune regulation. Unregulated bioactivity of these cytokines can lead to tissue-damaging inflammation^34^. A number of mechanisms such as proteolytic processing^15^,^16^,^17^, receptor antagonists and soluble receptors^18^ have evolved to regulate their activities. Consistent with its proposed ‘alarmin’ role we detected release of IL-33 in the lung of asthmatics almost exclusively during disease exacerbation. In addition, we present here a mechanism for the rapid termination of IL-33 activity at its receptor ST2 through a conformational switch, driven by oxidation and bridging of the four free cysteines in IL-33. Disruption of this inactivation mechanism *in vivo* leads to a profound enhancement of inflammation. Interestingly, several other IL-1 family members contain free cysteine residues in their IL-1-like cytokine domains: two in IL-1β, four in IL-18, four in IL-36α, six in IL-36β, and four in IL-36γ, while IL-1α has a single free cysteine. Of these cytokines, only IL-18 has previously been suggested to be susceptible to oxidation^35^ although this was not studied in detail. In fact, our data suggest that, under conditions that promote disulphide bond formation in IL-33, all but IL-1α and IL-36β show evidence of oxidative changes. It is tempting to speculate that cysteine oxidation could be an important mechanism utilised by a number of IL-1 family members to regulate their activity and systemic exposure.

## Methods

### Mice

WT BALB/c mice were purchased from Charles River, UK. hIL-33^*+/+*^/mIL-33^*−/−*^ (humanized IL-33) mice were generated in collaboration with TaconicArtemis GmbH (Supplementary Fig. 12). Mouse genomic fragments (obtained from the C57BL/6J RPCIB-731 BAC library), a human genomic fragment (obtained from the human RPCIB-753 BAC library) and selected features (such as recombination sites and selection markers) were combined to make the targeting vector, which was transfected into a BALB/c ES cell line. Clones resistant to both Puromycin (positive selection) and Gancyclovir (negative selection) were isolated and screened extensively to identify ones having undergone homologous recombination. Correctly targeted ES clones were microinjected into C57BL/6 host blastocysts and transferred to pseudopregnant females. The ES cell contribution in the resultant chimaeras (G0) was determined by coat colour (white/black). Chimeric mice with a high degree of ES contribution were bred to strain BALB/cJBomTac females carrying a Flp recombinase gene (Flp-Deleter strain) to generate G1 offspring. Germline transmission was confirmed by PCR genotyping using primers specific for the Flp-recombined targeted allele. Humanized IL-33 mice were bred and maintained under specific pathogen-free conditions in accredited animal facilities at MRC-Harwell, UK. Male or female WT or humanized IL-33 mice (6–10 weeks old) were anaesthetized briefly with isofluorane and administered either 25 μg of ALT extract (Greer, Lenoir, NC), recombinant IL-33 protein or vehicle intranasally in a total volume of 50 μl. At selected timepoints after challenge, mice were terminally anaesthetised with pentobarbital sodium. Bronchoalveolar lavage fluid (BALF) was collected by lavage with PBS (0.3 ml, 0.3 ml and 0.4 ml) via tracheal cannula. BALF was centrifuged, cells counted and cell free supernatant was analysed for cytokines (Meso Scale Discovery, Rockville, MD). Differential cell counts (200 cells per slide) were performed on cytospin preparations stained with Diff-Quik (Fisher Scientific,UK). Rarely, animals that appeared to have been mis-dosed and clear technical failures were excluded from analyses. All work was carried out to UK Home Office ethical and husbandry standards under the authority of an appropriate Project Licence, which was approved by Babraham Institute Animal Welfare and Ethical Review Body (AWERB). In the data presented the group sizes were set in order to generate sufficient material for *ex vivo* analyses and determine profiles of IL-33 forms. For inflammation studies, sample size analysis was performed using previous experimental data. Randomization was performed at the time of animal delivery and although formal blinding was not used, the dosing and sample collection was performed by technical staff not involved in protein generation or analysis.

### Recombinant proteins

IL-33 proteins were purchased (Adipogen (human); R&D Systems, Peprotech (mouse)) or generated in-house. Human IL-33 (amino acids 112–270 (accession number Swiss-Prot O95760)) or mouse IL-33 (amino acids 102–166 (accession number Swiss-Prot Q8BVZ5) was modified to contain a FLAG 10 × His epitope tag (DYKDDDDKAAHHHHHHHHHH) at the C-terminus of the protein or 10 × His, Avitag and Factor-Xa protease cleavage site (MHHHHHHHHHHAAGLNDIFEAQKIEWHEAAIEGR) at the N terminus of the protein. Proteins were expressed in *Escherichia coli* and purified via nickel affinity chromatography and size exclusion chromatography (SEC). For untagged IL-33, N-terminal tagged protein was Factor Xa cleaved and repurified. IL-33 cytokine trap for human IL-33 was designed based on Economides *et al*.^36^ and consisted of amino acids 1–359 (Swiss Prot accession number Q9NPH3) fused to amino acids 19–328 (Swiss Prot accession number Q01638) fused to the Fc portion of human IgG_1_. Human IgG_1_ antibodies were generated in CHO cells. Proteins were biotinylated via free amines using EZ link Sulfo-NHS-LC-Biotin (Thermo/Pierce) or via free cysteines using EZ link Biotin-BMCC (Perbio/Pierce). Human ST2.Fc, IL-1 and IL-36 proteins were purchased (R&D systems). Human IL-18 (Uniprot entry Q1411) was modified to contain an N-terminal tag with a Factor Xa cleavage site and was expressed and purified in the same way as IL-33 followed by removal of the tag with Factor Xa.

### Disulphide bonded IL-33

Human IL-33 was incubated with 60% IMDM media at a final protein concentration of 300 μg ml^–1^ for 18 h at 37 °C. After 18 h, media-treated IL-33 was purified from media components using SEC on an S75 16:600 Superdex column (GE Healthcare) in 2 × DPBS using an AKTAxpress FPLC system (GE Healthcare). Peak fractions were analysed by SDS–PAGE. For NMR studies, purified protein was further concentrated to 1.8 mg ml^−1^ and analysed by SDS–PAGE.

### IL-33 detection reagents

IL-33 antibodies were isolated by phage display (IL330004, IL330425, 640050) or purchased from commercial suppliers (R&D Systems #AF3625, #AF3626). IL-33 was quantified by Duoset ELISA (R&D Systems #DY3625, #DY3626), Luminex assay (Millipore #HTH17MAG-14K), according to manufacturer’s instructions, or in house ELISAs where IL330425 (150 μg ml^−1^), IL330004 (150 μg ml^−1^) or 640050 (2 μg ml^−1^) were used as capture antibodies. Captured IL-33 was detected with biotinylated sST2.Fc (R&D Systems #523-ST-100, 1 μg ml^−1^), biotinylated IL330425 (1 μg ml^−1^) or biotinylated goat polyclonal (R&D Systems #BAF3625, 500 ng ml^−1^), respectively.

### SDS–PAGE and western blot analysis

IL-33 was analysed by SDS–PAGE on NuPAGE Novex 12% Bis-Tris mini gels (Invitrogen) with MOPS running buffer (Invitrogen) according to manufacturer’s instructions under reducing or non-reducing conditions. Reduced samples contained 2% beta-mercaptoethanol. Gels were stained with Coomassie brilliant blue G-250 based gel staining reagent (Sigma). Alternatively, proteins were transferred to nitrocellulose membranes (Invitrogen) and detected by western blotting with goat anti-IL-33 pAb (R&D systems #AF3625, #AF3626) at 1:1,000 dilution. Images have been cropped for presentation. Full size images are presented in Supplementary Figs 1, 3, 11, 13 and 17.

### Mass spectrometry

Reverse phase LC-MS analysis was performed using an Acquity UPLC coupled to a Synapt G1 quadrupole time of flight (QTOF) mass spectrometer (Waters, Milford, USS). Two micrograms of purified protein diluted in 10 mM Tris HCl pH 8 at 1 mg ml^−1^ was injected onto a 50 × 2.1 mm, 1.7-μm particle size BEH C4 analytical column held at 65 °C (Waters, Milford,USA). Protein was eluted at a constant flow rate of 0.15 ml min^−1^ using a 5-min binary gradient; solvent B was initially increased from 5 to 95% over 1 min, reduced to 20% over 2 min and returned to 5% over a further 2 min. The column was cleaned before the next injection by oscillating between high (95%) and low (5%) solvent B for 5 min. Solvent A (water) and B (acetonitrile) were supplemented with 0.01% (v/v) trifluoroacetic acid and 0.1% (v/v) formic acid. Spectra were acquired between 500–4,500 m/z. Key instrument parameters included +ve ionisation mode, source voltage: 3.4 kV, sample cone voltage: 50 V, source temperature: 140 °C, desolvation temperature: 400 °C.

### Disulphide mapping

For each sample, 50 μg of protein was prepared at 3 mg ml^–1^ in 100 mM sodium phosphate, 1 mM *N*-ethylmaleimide, pH 7.0 buffer and incubated for 20 min at room temperature. Dried samples were resuspended in 7 M Guanidine HCl, 100 mM NaCl, 10 mM sodium phosphate and incubated at 37 °C for 30 min. Denatured protein was diluted to 0.3 mg ml^–1^ and digested with Lys-C at an E:S ratio of 1:50 in 2 M guanadine, 100 mM sodium phosphate, 0.1 mM EDTA, pH7.0 at 37 °C. After 2 h a second, equal aliquot of Lys-C was added. After a further 2 h the digest was split; for the reduced analysis the digest was incubated with 50 mM dithiothreitol for 15 min at room temperature. Reduced and non-reduced samples were analysed by RP LC-MS using an Acquity UPLC coupled to a Synapt G2 QToF mass spectrometer (Waters, Milford, USA). For each sample, 5 ug of Lys-C digest was injected onto a 150 × 2.1 mm, 1.7 μm particle size BEH300 C18 analytical column held at 55 °C (Waters, Milford,USA). Peptides were eluted at a constant flow rate of 0.2 mL min^–1^ using a 75 min binary gradient; solvent B was increased from 0 to 35%. The column was cleaned before the subsequent injection by oscillating between high (95%) and low (5%) solvent B for 5 min. Solvent A (water) and B (acetonitrile) were supplemented with 0.02% (v/v) trifluoroacetic acid. Spectra were acquired between 50–2,000 m/z using a data independent mode of acquisition. Low and high energy spectra were processed using BioPharmaLynx (Waters, Milford, USA).

### NMR

The human IL-33^112–270^ sequence was modified to include an N-terminal 6His tag and TEV protease cleavage site (MGHHHHHHGGGENLYFQGS). IL-33 proteins were produced in the presence of M9 minimal media supplemented with 5 g l^–1^ of ^15^N -IsoGro powder and detagged with TEV protease. NMR spectra were recorded at 298 K on a Bruker Avance 600 MHz spectrometer running Topspin 2.3 equipped with a 5 mm TCI Cryoprobe with Z-axis gradients. The ^15^N-labelled IL-33 WT sample was prepared as described with the addition of 5% deuterium oxide to allow sample locking. ^1^H–^15^N correlation spectra were acquired employing the sofast HMQC pulse sequence^37^ with (F2xF1) 1,024 × 64 complex points (in states-TPPI mode), 9,615 × 1,460 Hz sweep width, 53.4 × 43.8 ms acquisition times.

### Circular dichroism (CD) spectroscopy

Far-ultraviolet and near-ultraviolet CD analysis were performed on a Jasco-815 instrument (Easton, Maryland). For Far- ultraviolet CD, spectra were recorded over wavelength range 180–260 nm in a 1-mm pathlength cuvette at sample concentration of 0.14 mg ml^−1^ and 0.12 mg ml^−1^ for IL-33 and DSB IL-33, respectively, at 20 °C in buffer solution 10 mM Phosphate pH 6.9. For near-ultraviolet CD, spectra were recorded over wavelength range 260–350 nm in a 10-mm pathlength cuvette at sample concentration of 1.38 mg ml^−1^ and 0.89 mg ml^−1^ for IL-33 and DSB IL-33, respectively, at 20 °C in buffer solution DPBS. CD spectra of the buffer solution were recorded and subtracted from all sample spectra to correct for instrument, cuvette and baseline effects. Aromatic amino acids and disulphide absorption bands are adapted from Kelly *et al*^38^.

### Hydrogen-exchange mass spectrometry (HX-MS)

Proteins were diluted to 3.5 μM in phosphate buffered saline, pH 7.4. This stock was used to initiate labelling experiments by diluting 10-fold with deuterated (10 mM sodium phosphate, pD 6.6) aqueous solvent. Initial mapping experiments were performed to assign mass spectra to peptic peptide sequences from IL-33. This was done largely as described^39^. Briefly, protonated diluted protein was mixed 1:1 with a quench solution (100 mM potassium phosphate, pH 2.55, 0.1 M TCEP, 1 °C), such that the final mixture pH was 2.55. The quenched protein was injected into a Waters HDX Manager with an immobilized pepsin column (2.0 × 30 mm; Poroszyme, Life Technologies), C18 trapping column (VanGuard ACQUITY BEH 2.1 × 5 mm; Waters) and analytical C18 column (1.0 × 100 mm ACQUITY BEH; Waters). Mobile phases were 0.1% formic acid in H_2_O (A) and 0.1% formic acid in acetonitrile (B), such that their pH was 2.55. Protein was applied to the pepsin and trapping columns in 100 μl min^–1^ buffer A and eluted from the analytical column in a linear gradient of 3–40% B at 40 μL min^–1^. Peptide sequences were assigned from MSE fragment data with Protein Lynx Global Server (Waters) 3.0.2 and DynamX 3.0 (Waters). Labeling data was acquired as for sequencing, except the mass spectrometer was set to MS scans only. Peptide-level data were analysed in DynamX and MatLab (Mathworks).

### ST2 binding measurements

Direct binding of IL-33 to the extracellular domain of ST2 was determined by surface plasmon resonance using a BIAcore 2000 (GE Healthcare). ST2 was immobilised using an anti-human Fc capture (GE Healthcare BR-1003-39) on a CM5 sensor chip (GE healthcare BR-1003-99) to give a stable surface of ∼150 RU. IL-33 was flowed over the surface at 30 μl min^–1^ for 3 min to determine association rates. Dissociation was measured by flowing buffer at 30 μl min^–1^ for 15 min. Sensorgrams were interpreted using BIAevaluation software and kinetics were determined using double reference subtracted sensorgrams using a 1:1 (Langmuir) binding model.

### Cell culture and bioassays

Normal human umblical vein cells (HUVECs) were obtained from Cambrex and maintained in EBM-2 culture media (Lonza) according to manufacturer’s protocol. Cells were confirmed negative for mycoplasma. Cells were stimulated with IL-33 for 30 min, fixed with formaldehyde and stained for NFkB p65 subunit (Thermo-Fisher) according to manufacturer’s protocol. Hoechst dye was used to stain the cell nuclei. Plates were analysed for nuclear NFkB p65 translocation using an ArrayScan VTi HCS Reader. Human cord blood-derived mast cells were produced by *in vitro* differentiation of cord blood CD133^+^ progenitor cells (Lonza) as described^40^. Cells were cultured in Serum Free Expansion Media (StemSpan) supplemented with Penicillin/ streptomycin (Invitrogen) and growth factors: 100 ng ml^−1^ Stem Cell Factor, 50 ng ml^−1^ IL-6 (Peprotech). 1 ng ml^−1^ IL-3 (R&D Systems) was included in the culture media during the first three weeks. For assays, IL-6 was removed from the culture media 24 h prior to stimulation with IL-33. IL-13 was measured in cell supernatants (Mesoscale Discovery) from human cord blood-derived mast cells stimulated with IL-33 overnight. Data were analysed using Graphpad Prism software. IC_50_ values were determined by curve fitting using a three or four-parameter logistic equation.

### Human tissue samples

Non-cancerous adjacent tissue from lung cancer patients and from lung transplant surgeries were supplied in Aqix RS-I medium (Aqix Ltd) on ice by Papworth Hospital NHS Trust Research Tissue Bank. The tissues used in this study were from COPD or asthmatic ex-smoker patients. ∼3 g of lung tissue was washed 3 × in PBS in a sterile Petradish (Costar) and ∼0.5 mm^2^ tissue explants prepared using a sterilised metal cork borer. Explants were incubated in PBS/0.1% BSA in wells of a 96-well tissue culture plate (Costar) for 15, 30 min, 1, 2, 5 and 20 h under standard tissue culture conditions. Each time point was performed with *n*=8 replicates and pooled supernatant samples from each time point were centrifuged (to remove any debris), aliquoted and snap frozen on dry ice. IL-33 forms in supernatants were quantified by ELISAs as described. The study was approved by the NRES East of England (Cambridge East) Research Ethics Committee (reference number 08/H0304/56+5) and tissue was donated with the informed consent of patients.

### Human sputum samples

Subjects with asthma were recruited from a single centre at the Glenfield Hospital, Leicester, United Kingdom. Assignment to severe asthma was made by the subjects’ physician consistent with definitions of asthma according to the Global Initiative for Asthma guidelines^41^. All subjects were clinically assessed at stable visits at least 8 weeks free from an exacerbation, defined as an increase in symptoms necessitating a course of oral corticosteroids and/or antibiotic therapy. All subjects provided written informed consent, and the studies were approved by the local Leicestershire, Northamptonshire, and Rutland ethics committee. Spontaneous or induced sputum was collected and mixed with 8 volumes of PBS. Cell-free sputum supernatant was used for mediator assessment.

### Primary biotinylation site mapping

Human IL-33 was –SH biotinylated with EZ link Biotin-BMCC (Pierce/Thermo 21900) in PBS using a IL-33:reagent molar ratio of 1:1. After buffer exchange into D-PBS, MS analysis indicated that 50% of the IL-33 material was in the native state and 50% was mono-biotinylated. Biotinylated IL-33 was captured on high capacity streptavidin agarose (HCSA) beads (Thermo/Pierce). The IL-33 beads were washed and incubated in PBS±diluted cell culture grade trypsin. After 10 min room temperature digestion, the beads were washed and spun to dryness. The recovered beads were exposed to a saturated solution of alpha-cyano-4-hydroxycinnamic acid (CHCA) in 50:50:0.5 acetonitrile:water:trifluoroacetic acid solution. This MS matrix solution was harsh enough to release detectable quantities of the streptavidin bead -bound biotinylated-IL-33 peptide material. one microlitre of the extracted liquid phase was then spotted onto a ABI4800 MALDI target plate, dried, and analysed using MALDI-TOF MS (ABI4800). A 2,496-Da proteolytic fragment dominated the MS spectra and this was chosen as a precursor ion for MS:MS fragmentation analysis in positive (1 kV) reflector mode. The mass of a single BMCC-biotin incorporation is 533 Da. A search of the known human IL-33 sequence using FindPep (expasy.org) was made assuming that the 2,496 Da precursor ion is composed of an IL-33 related peptide that has incorporated a single biotin modification, such that the peptide mass is 1,963 Da (2,496–533 Da).

### Epitope mapping H338L293 mAb

H338L293 IgG was complexed with IL-33 over several hours. Trypsin was added to pre-formed IL-33:H338L293 IgG complexes, followed by SEC analysis. Trypsin digest led to an increase in the retention time of the main peak to 14.1 min (intermediate between the untreated complex peak elution time (13.6 min) and the intact H338L292 IgG elution (14.4 min)). Mass spectrometry methods were then used to identify the minimal H338L293 IgG epitope. The Shimadzu MALDI-TOF MS observed masses from captured 14.1 min peak were 3,209 and 4,485.3 Da. ABI4800 MALDI-TOF MS observed masses were 3,208.9 Da peak at high intensity with a secondary 4,486.4 Da peak also present. The observed precursor ion mass of 3,206–3,208 Da and ABI4800 MS/MS fragmentation analysis of the 3,206 Da precursor ions matched the predicted tryptic IL-33 fragment MLMVTLSPTKDFWLHANNKEHSVELHK. The identified peptide together with a truncate (LSPTKDFWLHANNKEHSVELHK) and scrambled variants of both, were chemically synthesised and used in confirmatory T100 Biacore (GE Healthcare) binding studies.

### Molecular dynamics

Classical molecular dynamics simulations of wild type IL-33 in solution were conducted via GROMACS^42^ software package (version 4.6.5). Starting from solvating the first frame of the NMR structure (PDB code: 2KLL) and counter ions (Na+) into a water box with periodic boundary condition, the system was equilibrated at constant NVT and NPT (*P*=1 bar, T=300 K) environments with restraints on protein for both 100 ps, and then further equilibrated for 10 ns without restraints. 200 ns constant NPT production run was performed afterwards. Amber99sb-Ildn force field and TIP3P water model integrated in GROMACS package were adopted to calculate atomic interactions. A 2 fs timestep and the LINCS bond constraint algorithm were used in all the simulations. System temperature and pressure were maintained by turning on the Berendsen thermostat and Parrinello-Rahman pressure coupling method. The particle mesh Ewald method was used to compute long-range electrostatic interactions.

## Additional information

**How to cite this article:** Cohen, E. S. *et al*. Oxidation of the alarmin IL-33 regulates ST2-dependent inflammation. *Nat. Commun.* 6:8327 doi: 10.1038/ncomms9327 (2015).

## Supplementary Material

Supplementary Information Supplementary Figures 1-17, Supplementary Table 1 and Supplementary References
