# Supplementary material for: Oxidation of the alarmin IL-33 regulates ST2-dependent inflammation
Source: Nat Commun. 2015 Sep 14;6:8327. doi: 10.1038/ncomms9327 (PMC4579851; doi:10.1038/ncomms9327)
Supplement: Supplementary Information — Supplementary Figures 1-17, Supplementary Table 1 and Supplementary References [file ncomms9327-s1.pdf]

SUPPLEMENTARY FIGURES

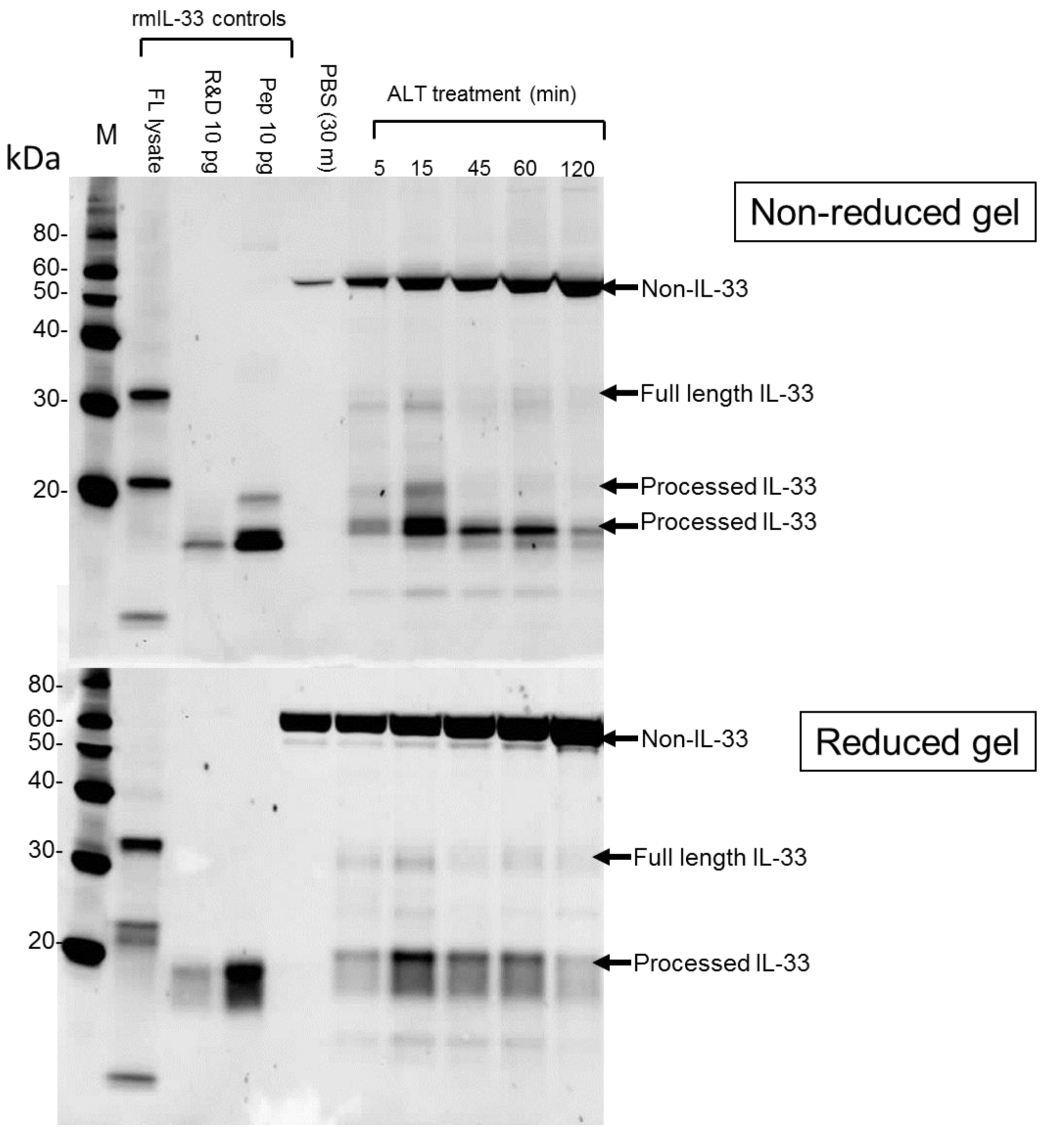

Supplementary Figure 1| Full blot of Figure 1b

Western blot analysis of bronchoalveolar lavage fluid (BALF) following intranasal *Alternaria* (ALT) challenge of BALB/c mice (n = 3 per group, pooled), under reducing and non-reducing conditions. Controls are as follows: cell lysate, lysate of HEK cells transfected with full length mouse IL-33; R&D, truncated mouse IL-33 (R&D systems); pep, truncated mouse IL-33 (Peprotech); PBS (30 min), BALF from vehicle (PBS) challenged mice at 30 min timepoint.

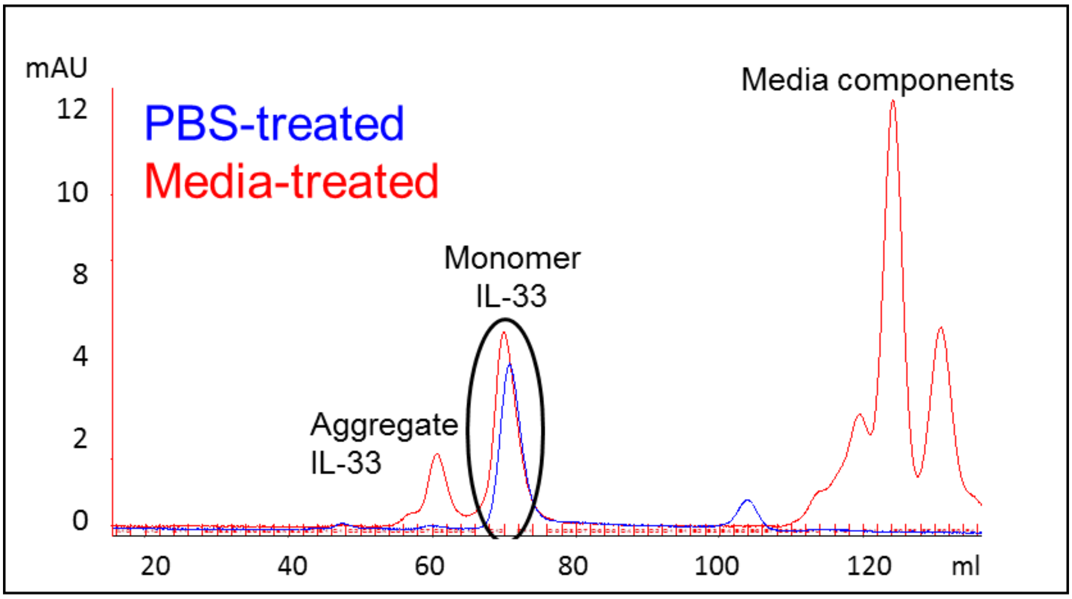

Supplementary Figure 2 | Purification of disulphide bonded IL-33.

Overlay of size exclusion chromatography traces for media-treated or PBS-treated human IL-$33^{112\text{-}270}$. The monomer fraction was collected for further analysis.

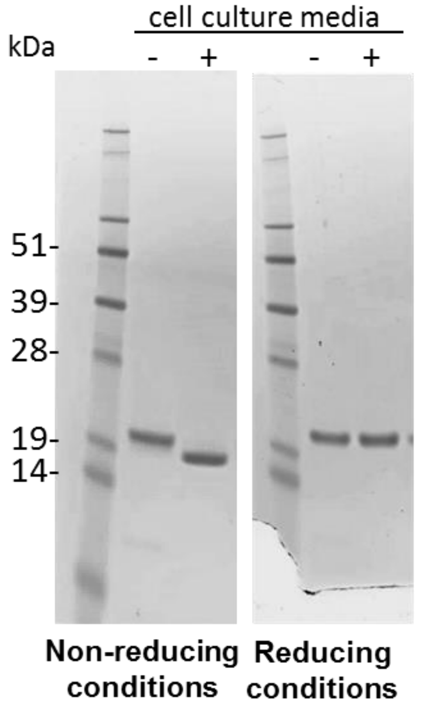

Supplementary Figure 3 | Full gel of Figure 1c

Non-reduced SDS-PAGE of IL-33$^{112-270}$ either untreated (-) or post treatment with cell culture media (Iscoves Modified Dulbeccos Media) (+). Monomer IL-33 was purified prior to analysis.

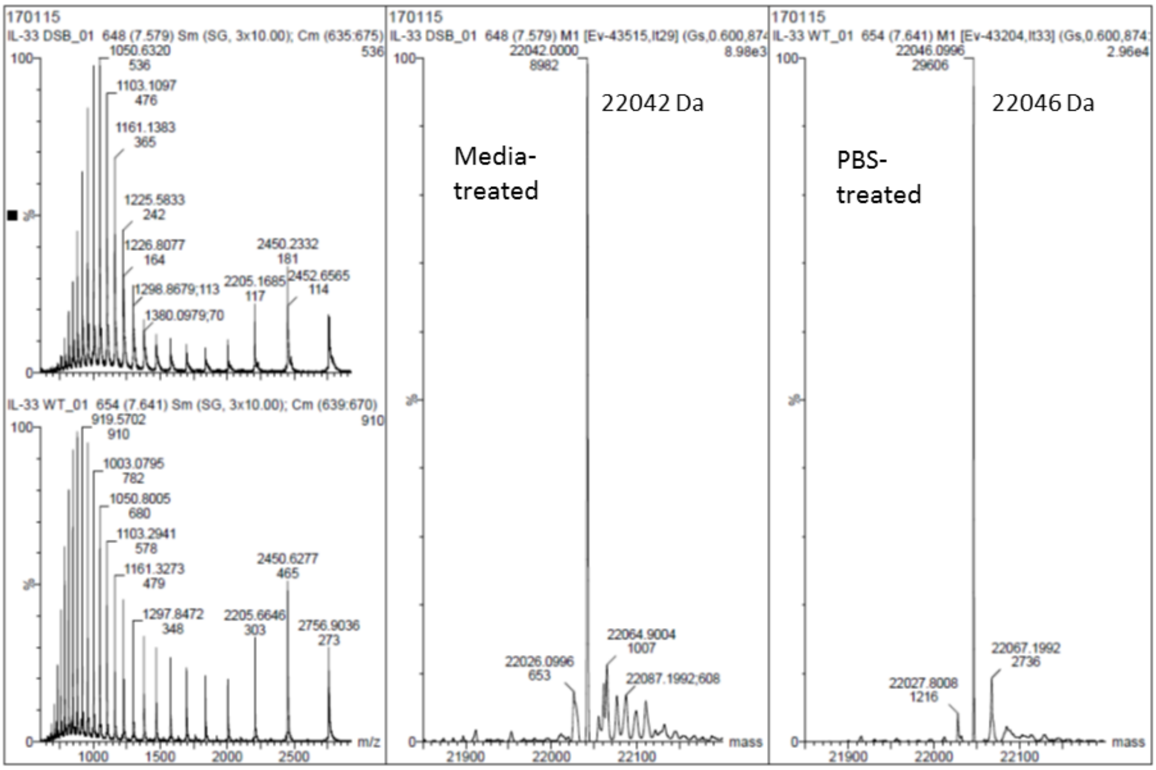

Supplementary Figure 4 | LC-MS analysis of media-treated IL-33.

Intact mass of PBS- versus media-treated N-terminal His-Avi tagged IL-$33^{112-270}$ determined by Reverse Phase (RP) LC-MS. 4 Da mass loss is observed after media treatment.

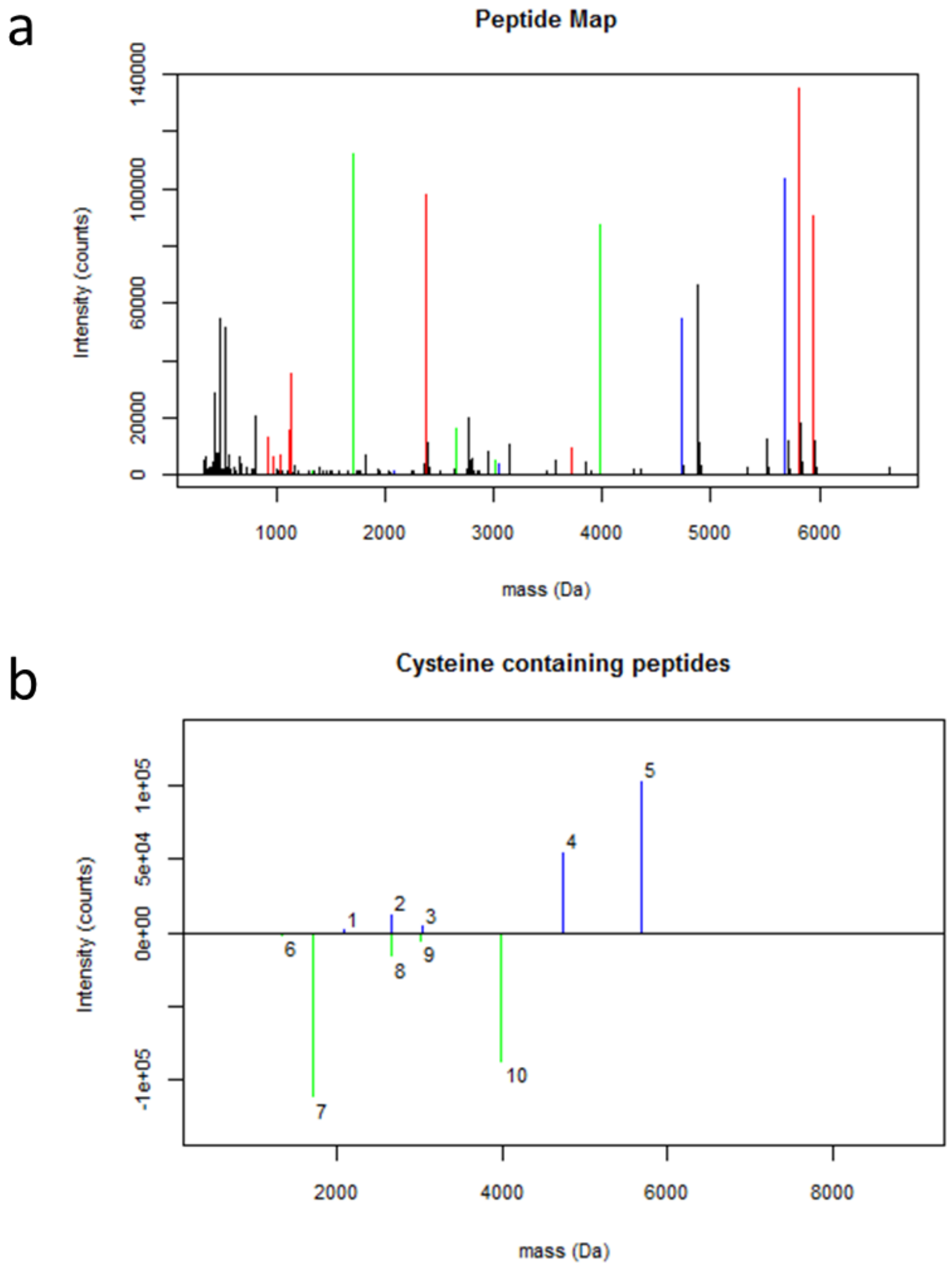

Supplementary Figure 5 | Disulphide mapping of media-treated human IL-33

a, Combined, deconvoluted mass spectra from non-reduced and reduced Lys-C peptide mapping analysis of DSB IL-33. **b**, Isolated spectra for cysteine containing peptides. Identified peptides common to reduced and non-reduced samples are highlighted in red; peptides unique to reduced and non-reduced samples are highlighted in green and blue, respectively. The sequences of the disulphide bonded peptides are described in Supplementary Table 1.

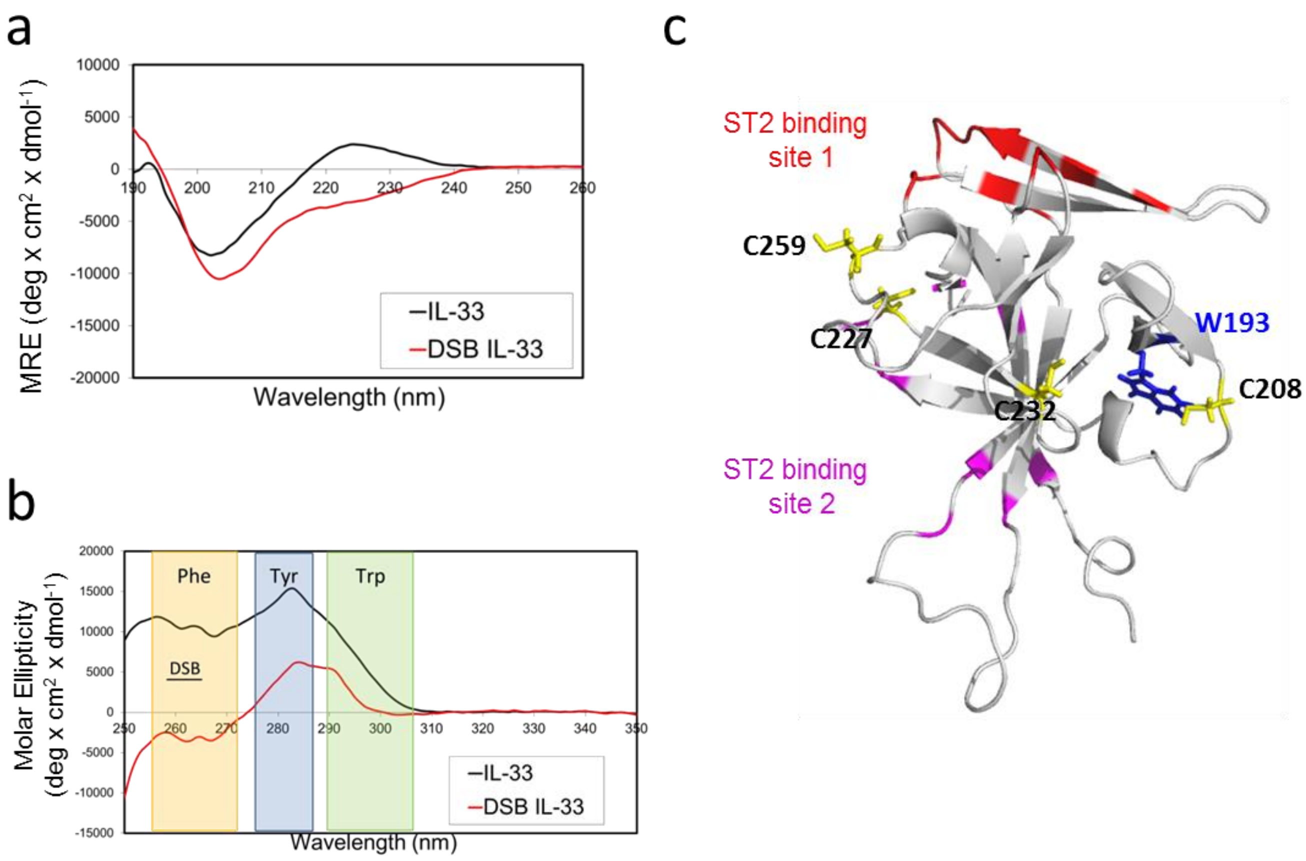

Supplementary Figure 6 | Circular dichroism spectroscopy of disulphide-bonded IL-33

a, Far-UV circular dichroism (CD) Spectroscopy. Spectra were recorded over wavelength range 190-260 nm. The final spectra were the average of 8 scans. **b**, Near-UV circular dichroism (CD) Spectroscopy. Spectra were recorded over wavelength range 260-350 nm. The final spectra were the average of 4 scans. Aromatic amino-acids and disulphide absorption bands are adapted from Kelly *et al.*[1] **c**, Key features of IL-33. Trp193, cysteines, and ST2 binding site[2] are indicated within the solved IL-33 structure.[3]

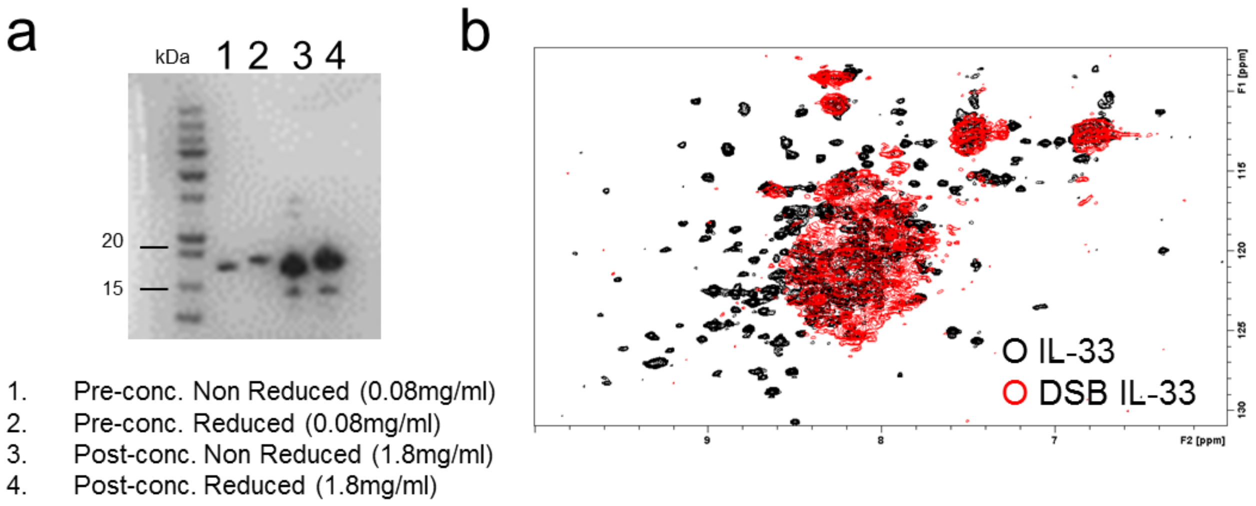

Supplementary Figure 7 | NMR analysis of disulphide-bonded IL-33

a, SDS PAGE analysis of human DSB IL33. Purified DSB IL-33 was analysed under reducing and non-reducing conditions before and after concentration for NMR. **b**, NMR heteronuclear multiple quantum coherence (HMQC) analysis with overlay of the ^{1}H-^{15}N HMQC spectra for ^{15}N-labeled human IL-33 for normal and disulphide bonded (DSB) forms. Spectra are significantly different and show a loss of the well-ordered, folded protein peaks between reduced IL-33 and DSB IL-33 respectively.

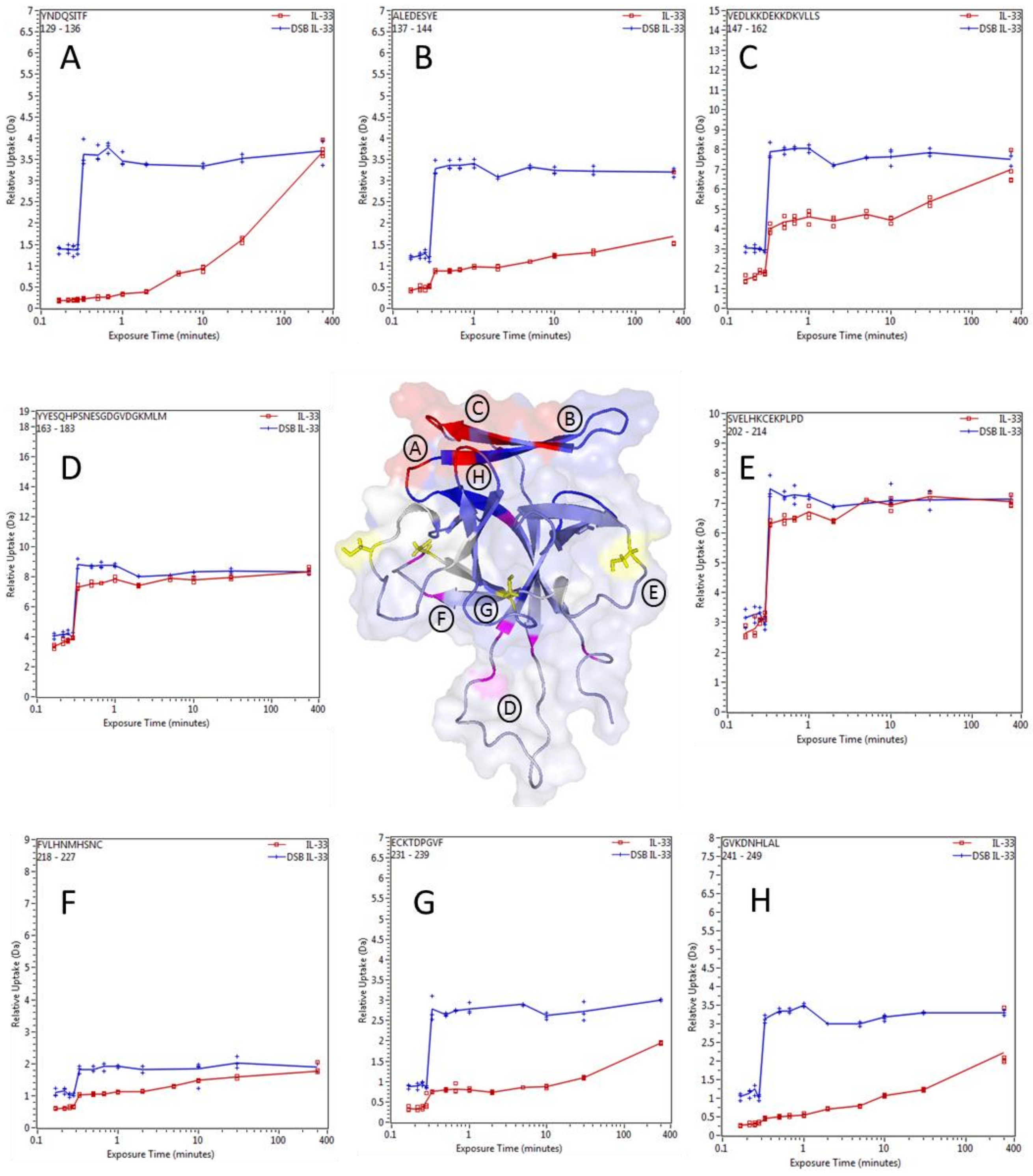

Supplementary Fig. 8 | Hydrogen exchange analysis of IL-33

Deuterium uptake plots for selected peptides of IL-33 and DSB IL-33. Location of peptides is indicated within the structural model of IL-33. Model depicts the regions of increased hydrogen exchange (dark blue) for DSB IL-33 versus IL-33 and is overlaid with the ST2 binding sites[2] (red and magenta) and cysteines (yellow).

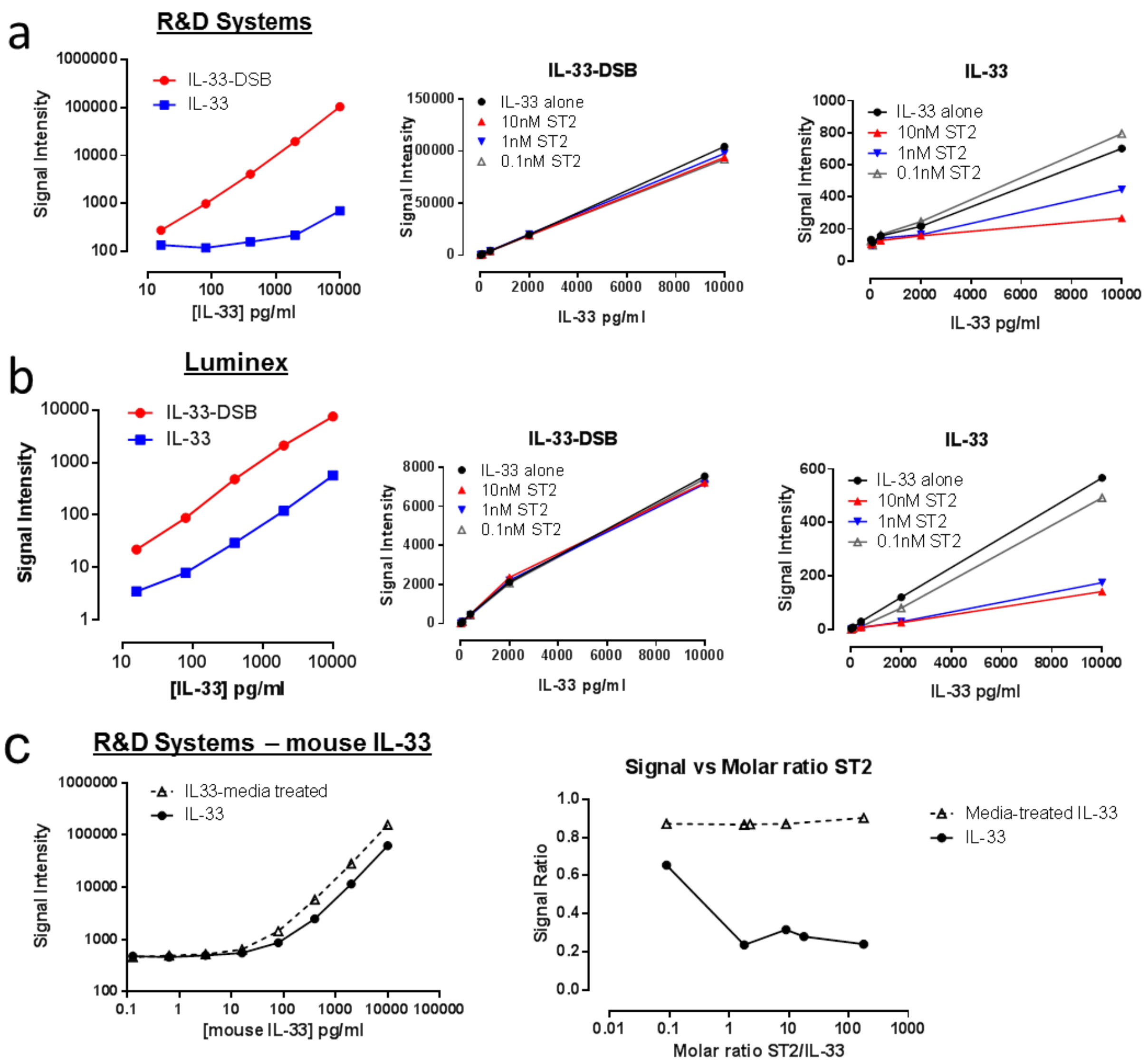

Supplementary Figure 9 | Validation of commercial ELISAs for IL-33

a, R&D systems commercial ELISA #DY3625; **b**, Millipore Luminex assay #HTH17MAG-14K. Left panel, standard curves of reduced and disulphide bonded (DSB) IL-33. Middle panel, DSB IL-33 signal is not affected by the presence of ST2.Fc. Right panel, reduced IL-33 signal is diminished by the presence of ST2.Fc. **c**, Validation of R&D systems commercial ELISA #DY3626 for detection of reduced and media-treated forms of mouse IL-33. Left panel, standard curves of reduced and media-treated IL-33; Right panel, Reduced IL-33 signal but not media-treated IL-33 signal is diminished in the presence of molar excess of ST2.Fc

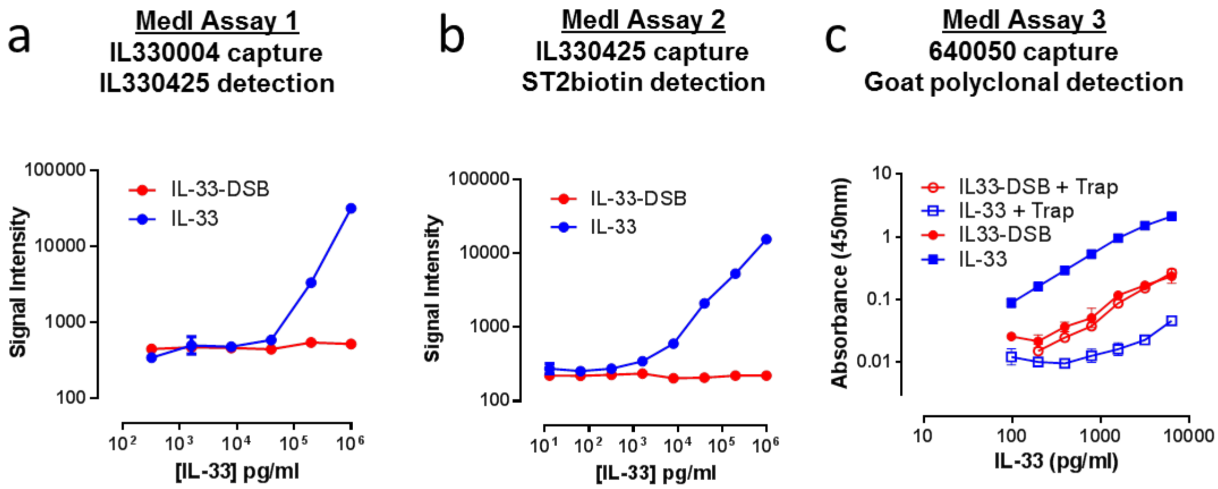

Supplementary Figure 10 | Development of ELISAs for reduced IL-33

a, **b**, In house assays specific for reduced IL-33. DSB IL-33 was not detected. **c**, In house ELISA that predominantly detects reduced IL-33. Signal with reduced IL-33 but not DSB IL-33 is diminished in the presence of molar excess of an IL-33 ST2/IL-1RAcP fusion protein (Trap).

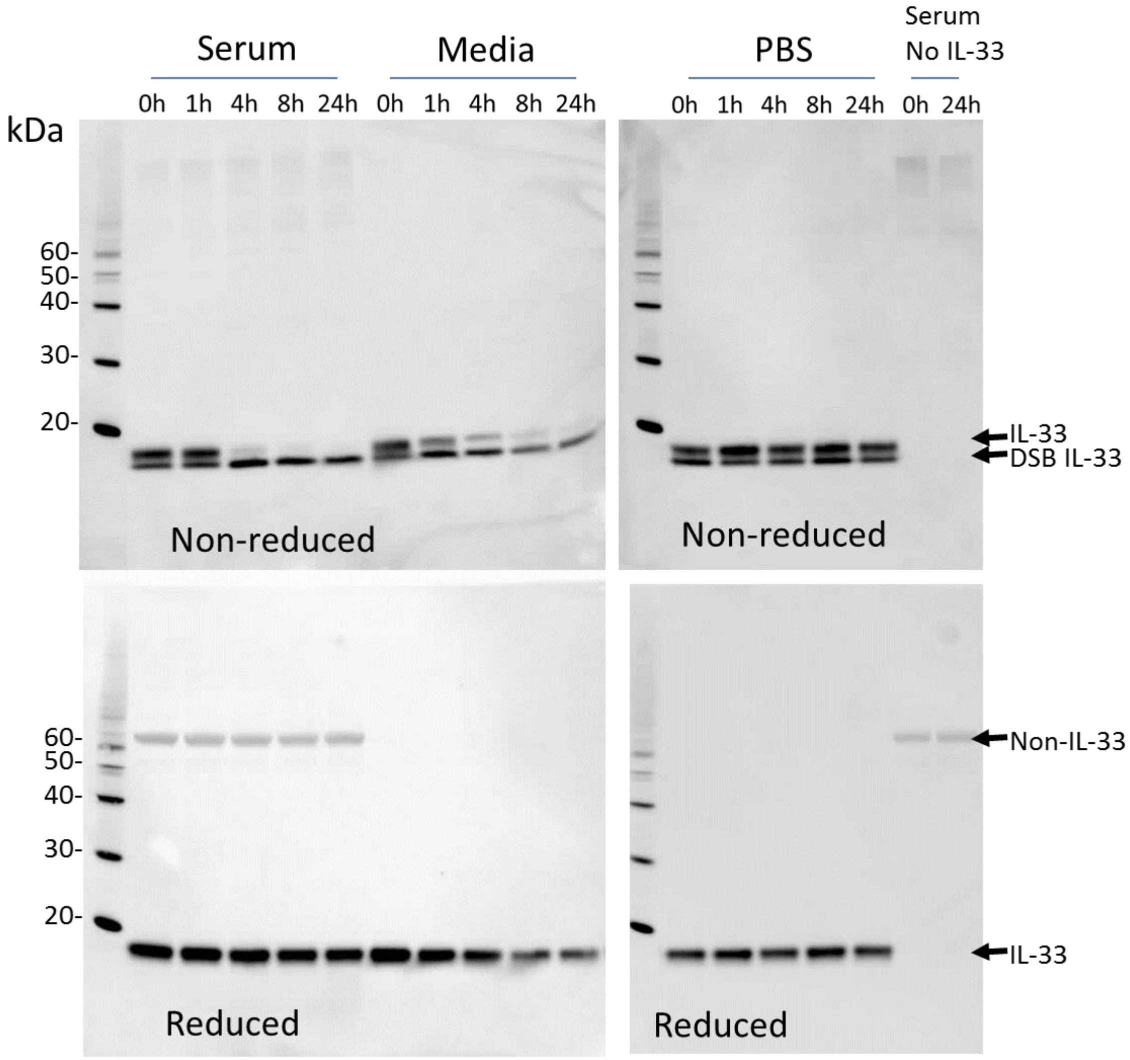

Supplementary Figure 11 | Full blot of Figure 2b

Change in human IL-33$^{112\text{-}270}$ after exposure to serum, cell culture media or PBS for varying time measured using western blot. Non-reducing conditions (top panels); reducing conditions (lower panels).

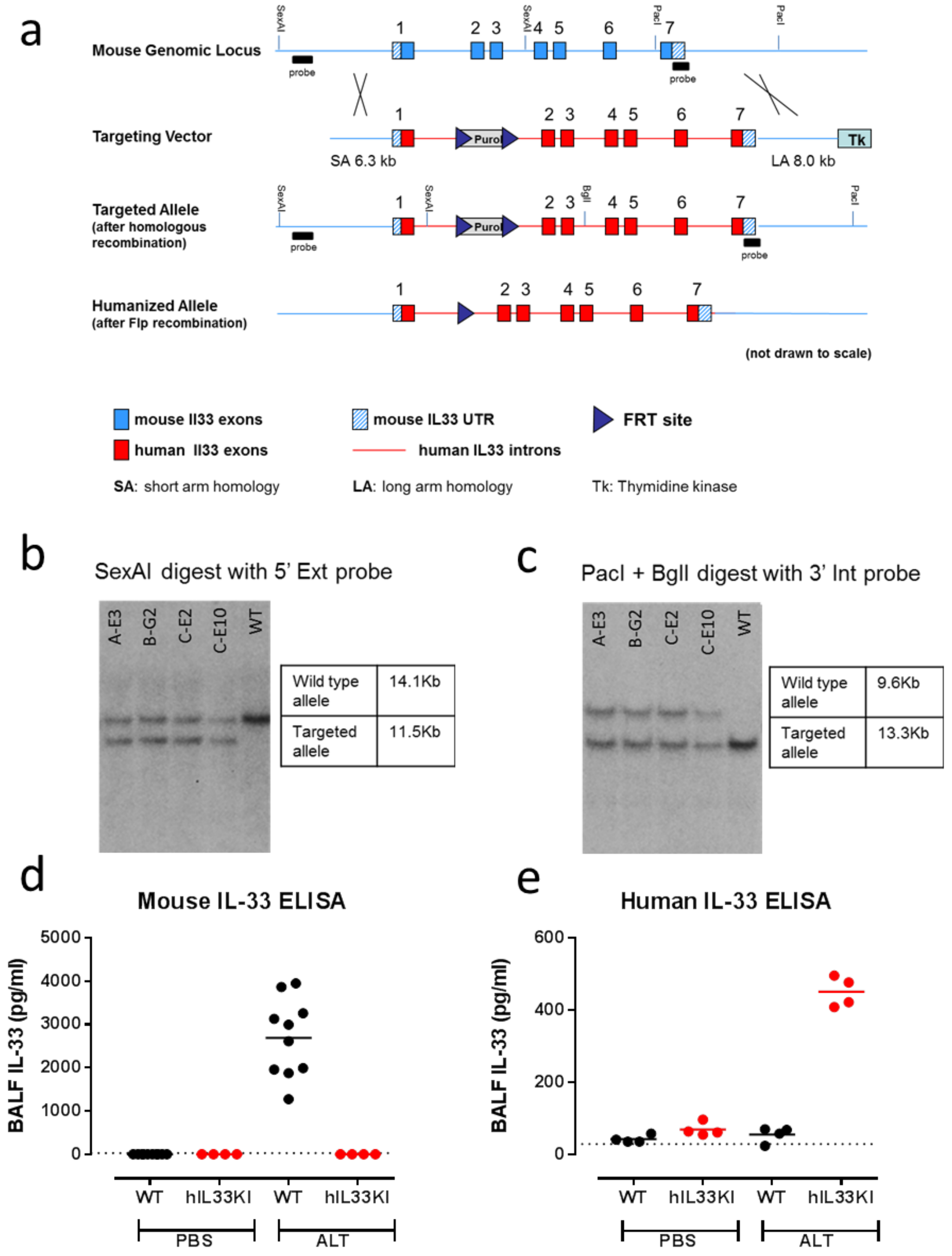

Supplementary Figure 12 | Generation of a humanized IL-33 transgenic mouse

a, Targeting vector. **b, c,** Southern blot data confirming correct 5' and 3' targeting in a selection of ES clones. Genomic DNA from the clones was digested with SexAI and probed with a 5' External probe (**b**) and a combination of PacI and BglI and probed with a 3' Internal probe (**c**). **d**, Detection of mouse IL-33 or **e**, human IL-33 in bronchoalveolar lavage fluid (BALF) following PBS or *Alternaria* challenge of wild type (WT) or humanized IL-33 mice. IL-33 was detected using commercial ELISAs (R&D Systems #DY3625, #AF3626) and quantified against the kit standards.

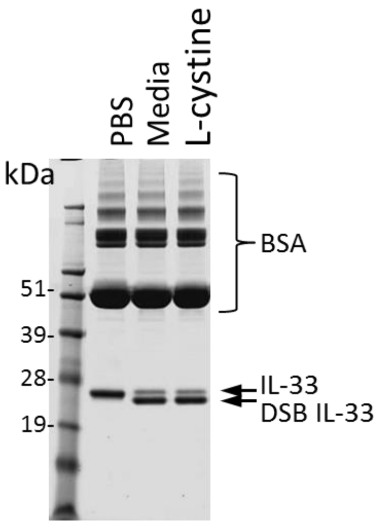

Supplementary Figure 13 | Full gel of Figure 3a

Non-reduced SDS-PAGE of N-terminal His-Avi tagged human IL-33$^{112\text{-}270}$ following overnight treatment in PBS+0.1%BSA, cell culture media+0.1%BSA, or 0.381mM L-cystine in PBS+0.1%BSA.

```
         192             208                 227  232                        259        270
          |               |                   |    |                          |          |
IL33-01 FWLHANNKEHSVELHKCEKPLPDQAFFVLHNMHSNCVSFECKTDPGVFIGVKDNHLALIKVDSSENLCTENILFKLSET
IL33-02 FWLHANNKEHSVELHKSEKPLPDQAFFVLHNMHSNCVSFECKTDPGVFIGVKDNHLALIKVDSSENLCTENILFKLSET
IL33-03 FWLHANNKEHSVELHKCEKPLPDQAFFVLHNMHSNSVSFECKTDPGVFIGVKDNHLALIKVDSSENLCTENILFKLSET
IL33-04 FWLHANNKEHSVELHKCEKPLPDQAFFVLHNMHSNCVSFESKTDPGVFIGVKDNHLALIKVDSSENLCTENILFKLSET
IL33-05 FWLHANNKEHSVELHKCEKPLPDQAFFVLHNMHSNCVSFECKTDPGVFIGVKDNHLALIKVDSSENLSTENILFKLSET
IL33-06 FWLHANNKEHSVELHKSEKPLPDQAFFVLHNMHSNSVSFECKTDPGVFIGVKDNHLALIKVDSSENLCTENILFKLSET
IL33-07 FWLHANNKEHSVELHKCEKPLPDQAFFVLHNMHSNCVSFESKTDPGVFIGVKDNHLALIKVDSSENLSTENILFKLSET
IL33-08 FWLHANNKEHSVELHKSEKPLPDQAFFVLHNMHSNCVSFESKTDPGVFIGVKDNHLALIKVDSSENLCTENILFKLSET
IL33-09 FWLHANNKEHSVELHKCEKPLPDQAFFVLHNMHSNSVSFECKTDPGVFIGVKDNHLALIKVDSSENLCTENILFKLSET
IL33-10 FWLHANNKEHSVELHKCEKPLPDQAFFVLHNMHSNSVSFESKTDPGVFIGVKDNHLALIKVDSSENLSTENILFKLSET
IL33-11 FWLHANNKEHSVELHKSEKPLPDQAFFVLHNMHSNCVSFECKTDPGVFIGVKDNHLALIKVDSSENLSTENILFKLSET
IL33-12 FWLHANNKEHSVELHKSEKPLPDQAFFVLHNMHSNSVSFESKTDPGVFIGVKDNHLALIKVDSSENLCTENILFKLSET
IL33-13 FWLHANNKEHSVELHKSEKPLPDQAFFVLHNMHSNSVSFECKTDPGVFIGVKDNHLALIKVDSSENLSTENILFKLSET
IL33-14 FWLHANNKEHSVELHKSEKPLPDQAFFVLHNMHSNCVSFESKTDPGVFIGVKDNHLALIKVDSSENLSTENILFKLSET
IL33-15 FWLHANNKEHSVELHKCEKPLPDQAFFVLHNMHSNSVSFESKTDPGVFIGVKDNHLALIKVDSSENLSTENILFKLSET
IL33-16 FWLHANNKEHSVELHKSEKPLPDQAFFVLHNMHSNSVSFESKTDPGVFIGVKDNHLALIKVDSSENLSTENILFKLSET
```

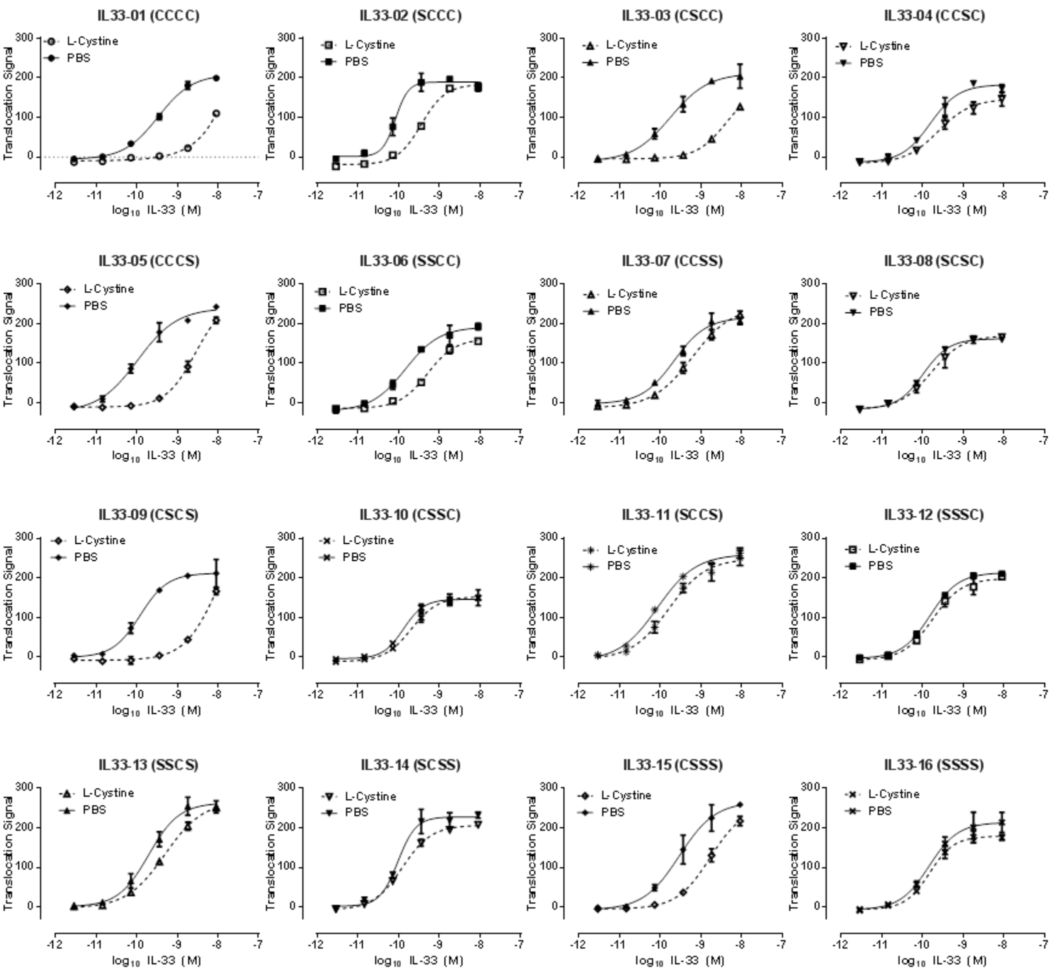

Supplementary Figure 14 | Activity of IL-33 Cys→Ser mutant panel

Activity was measured by stimulation of human umbilical vein endothelial cells (HUVEC) for 30 minutes and immunofluorescence detection of NFkB p65 nuclear translocation. Each graph shows an individual IL-33 protein before and after treatment for 18 hours with IMDM. Wild type IL-33 (IL-33-01), C208/C227/C232/C259 (CCCC) that has been pre-treated with culture media completely lost detectable activity. Mutants IL33-02 to IL33-16 have Cys→ Ser mutations at the positions indicated. All mutants were partially, moderately or completely protected from inactivation compared with WT.

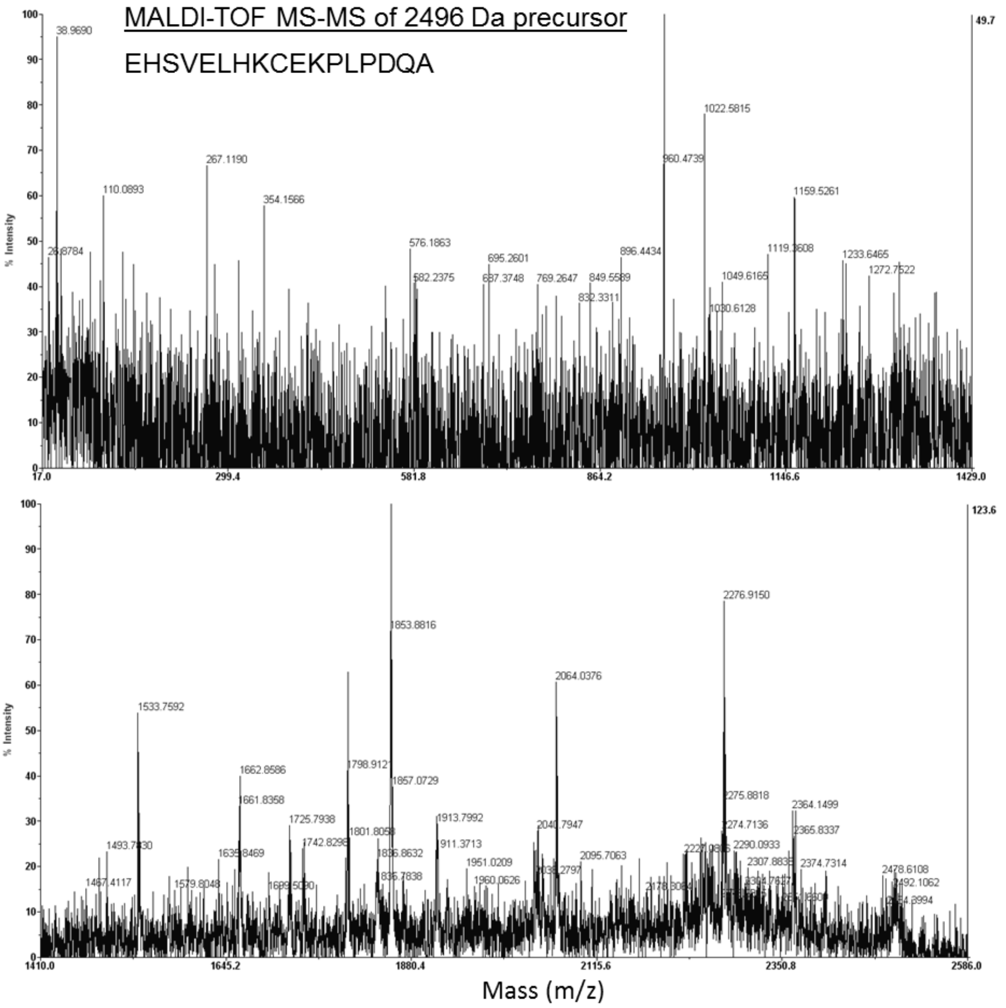

biotin	Observed mass	b-ions	Sequence	y-ions	Observed mass	biotin
		-	E	-		
	267.119	267.109	H	1830.917	2364.6	+ 533
	354.157	354.141	S	1693.858		
		453.209	V	1606.826		
	582.238	582.252	E	1507.757	2040.8	+ 533
		695.336	L	1378.715	1911.7	+ 533
	832.331	832.395	H	1265.631	1798.9	+ 533
	960.474	960.490	K	1128.572	1661.8	+ 533
		1063.499	C	1000.477	1533.7	+ 533
**+ 533**	1725.5	1192.541	E	897.468		
**+ 533**	1853.8	1320.637	K	768.425		
		1417.689	P	640.330		
**+ 533**	2063.8	1530.773	L	543.277		
		1627.826	P	430.193		
**+ 533**	2275.9	1742.853	D	333.141		
		1870.912	Q	218.114		
		-	A	90.055		

Supplementary Figure 15 | Mapping of IL-33 primary biotinylation site. Figure shows MALDI-TOF MS-MS analysis of a 2496 Da proteolytic fragment. –SH biotinylated IL-33 was captured on streptavidin beads and digested with trypsin. Biotinylated IL-33 peptide was released from the beads with alpha-cyano-4-hydroxycinnamic acid in 50:50:0.5 acetonitrile:water:trifluoroacetic acid solution and analyzed by MALDI-TOF MS. A 2496 Da proteolytic fragment dominated the MS spectra and was chosen as a precursor ion for MS:MS fragmentation analysis.

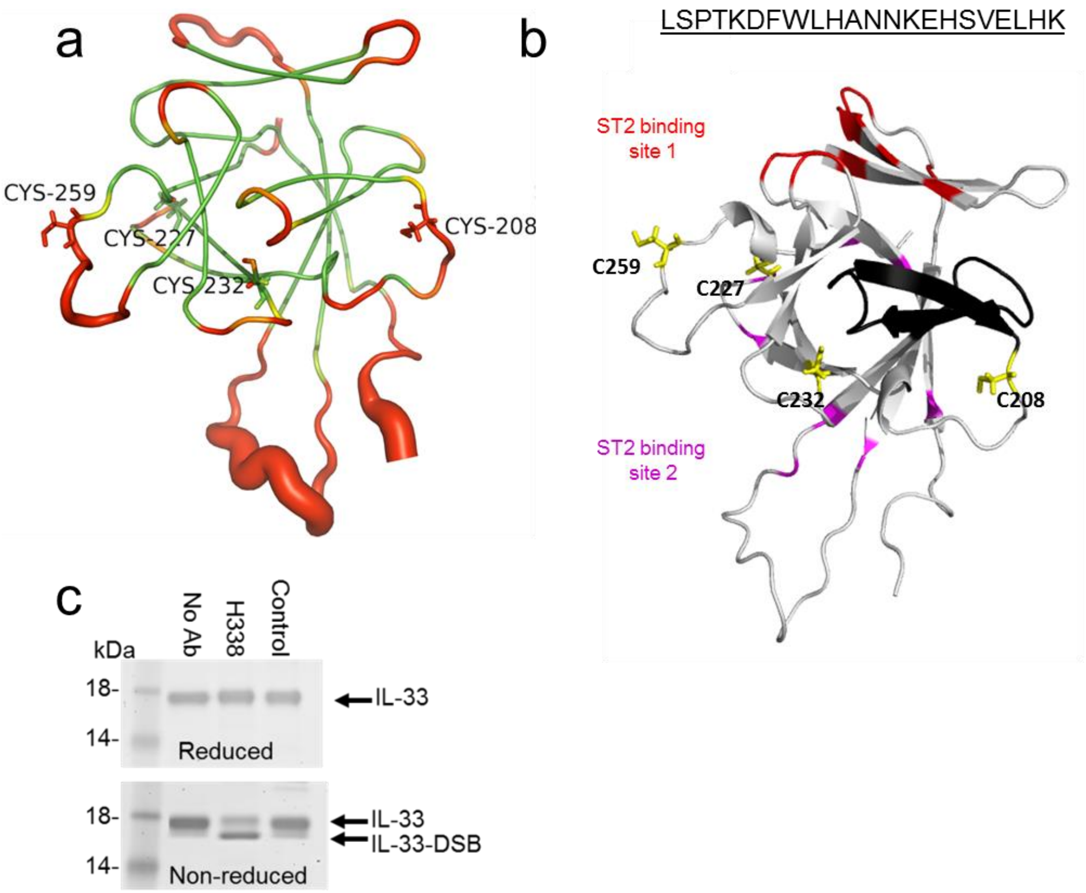

Supplementary Figure 16 | Structural dynamics of IL-33

a, Molecular dynamics simulations of wild type (WT) IL-33. Residue B factors, derived from structure root mean square fluctuation (RMSF) calculation over the entire simulation, were mapped on the original NMR structure[3] in the green-yellow-red scheme. Residues with B factor being less than 30 were coloured in green, and B factor being more than 60 were coloured in red. Any values in between were coloured in yellow. In WT IL-33 high structural fluctuations are observed for CYS-208. **b**, Location of IL-33 monoclonal antibody H338L293-binding region (black) within the published IL-33 structure.[3] The antibody epitope region does not overlap with the ST2 binding site (red and magenta).[2] **c,** Non-reducing SDS-PAGE analysis of human IL-33 incubated in PBS/0.1% BSA or in the presence of H338L293 or control mAb. Presence of the faster migrating, disulphide bonded (DSB) form of IL-33 is increased following incubation with H338L293 but not control mAb.

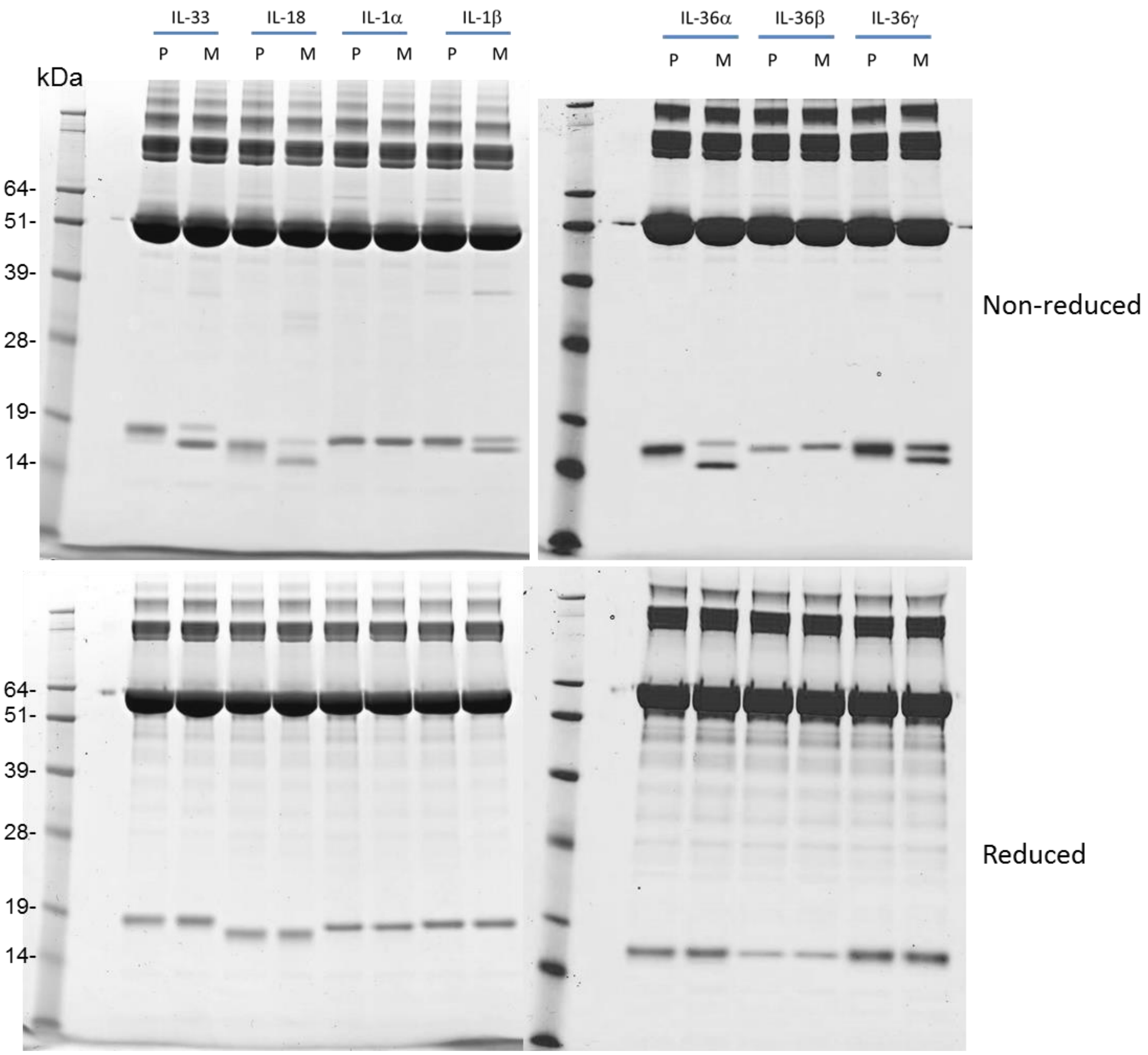

Supplementary Figure 17 | Full gel of Figure 4

SDS-PAGE of mature IL-1 family members following overnight treatment (P, PBS+0.1%BSA; M, cell culture media+0.1%BSA). Non-reducing conditions (top panels); reducing conditions (lower panel).

SUPPLEMENTARY TABLES

Peptides	Sequence	Cys oxidation status
	Non-reduced Lys-C peptide map	
1	C^{208}EK--VDSSENIC^{259}TENILFK	SS
2	PLPDQAFFVLHNMHSNC^{227}VSFEC^{232}K	SS
3	[EHSVELHK][C^{208}EK]--VDSSENIC^{259}TENILFK	SS
4	[C^{208}EK][PLPDQAFFVLHNMHSNC^{227}VSFEC^{232}K]--VDSSENIC^{259}TENILFK	SS
5	[EHSVELHK][C^{208}EK][PLPDQAFFVLHNMHSNC^{227}VSFEC^{232}K]--VDSSENIC^{259}TENILFK	SS
	Reduced Lys-C peptide map	
1	[EHSVELHK][C^{208}EK]	SH
2	VDSSENIC^{259}TENILFK	SH
3	PLPDQAFFVLHNMHSNC^{227}VSFEC^{232}K	SH
4	[C^{208}EK][PLPDQAFFVLHNMHSNC^{227}VSFEC^{232}K]	SH
5	[EHSVELHK][C^{208}EK][PLPDQAFFVLHNMHSNC^{227}VSFEC^{232}K]	SH

Supplementary Table 1 | Disulphide mapping. Sequences of disulphide bonded peptides identified by non-reduced and reduced Lys-C peptide mapping analysis of DSB IL-33 are shown. Disulphide linkages are represented by two hyphens (--). Lys-C miscleavages are represented by square brackets.

SUPPLEMENTARY REFERENCES

1. Kelly S. M. *et al*. How to study proteins by Circular Dichroism. *Biochimica et Biophysica Acta*, **1751**, 119-139 (2005)
2. Liu, X. *et al*. Structural insights into the interaction of IL-33 with its receptors. *Proc. Natl. Acad. Sci. U. S. A.* **110**, 14918-14923 (2013).
3. Lingel, A. *et al*. Structure of IL-33 and its interaction with the ST2 and IL-1RAcP receptors--insight into heterotrimeric IL-1 signaling complexes. *Structure* **17**, 1398-1410 (2009).
